# α2-3-sialylated glycosphingolipids in neuroinflammation, immunity, and programmed cell death: mechanistic evidence and context-dependent regulation: a comprehensive review

**DOI:** 10.3389/fimmu.2026.1865721

**Published:** 2026-08-19

**Authors:** Xiaocheng Li, Lu Li, Xinmeng Liu, Shujin Liu, Wanting Yang, Jiaxuan Gao, Shuxuan Kang, Lele Wang, Jiafan Li, Xingyu Wang, Haoqi Du, Shi Su, Zheng Li, Wen Xu

**Affiliations:** 1 Institute of Basic Medical Sciences, Xi’an Medical University, Xi’an, China; 2 Department of Electrophysiology, Weihai Municipal Hospital, Cheeloo College of Medicine, Shandong University, Weihai, Shandong, China; 3 Laboratory for Functional Glycomics, College of Life Sciences, Northwest University, Xi’an, China; 4 School of Medicine, Northwest University, Xi’an, China; 5 Department of Health Science Center, First Affiliated Hospital of Xi’an Jiao tong University, Xi’an, China

**Keywords:** immune regulation, lipid rafts, neuroinflammation, programmed cell death, α2-3-sialylated glycosphingolipids

## Abstract

α2-3-sialylated glycosphingolipids (α2-3-GSLs) are major constituents of neuronal membranes and lipid rafts, where they shape receptor compartmentalization, signal-complex assembly, and cell-cell communication. Their biological effects, however, vary by molecular subtype, cell type, disease stage, concentration, and local microenvironment. This review synthesizes evidence on spatiotemporal alterations in α2-3-GSL profiles and their relationships to neuroinflammation, immune responses, proteostasis, and programmed cell death across Parkinson’s disease, Alzheimer’s disease, Huntington’s disease, multiple sclerosis, and Guillain–Barré syndrome. Particular attention is given to GM1, GD1a, and GD3 and to mechanisms involving TLR4/NF-κB, PI3K/AKT, autophagy-lysosomal function, complement, damage-associated molecular patterns (DAMPs) recognition, and death-receptor signaling. Evidence is stratified into relatively well-supported, model-specific or incomplete, and conceptually inferred mechanisms. On this basis, we propose a lipid–inflammation–immunity–cell death framework that organizes potentially shared downstream processes while explicitly retaining disease-specific differences. This framework is not a validated universal causal pathway; rather, it provides an analytical structure for identifying evidence gaps and testable hypotheses. Disease-specific and parallel cross-disease studies, coupled with spatial lipidomics, *in vivo* tracing, and subtype-selective interventions, will be required to determine when α2-3-GSL manipulation is protective, neutral, or harmful and to support rational clinical translation.

## 1 Introduction

Glycosphingolipids (GSLs) are key components of neuronal cell membranes and consist of a hydrophobic sphingosine backbone covalently linked to a polar oligosaccharide chain through a glycosidic bond. Based on whether their oligosaccharide chains contain acidic groups (sialic acid or sulfate), GSLs are classified as neutral or acidic (1, 2). Gangliosides are the major subclass of acidic GSLs and are defined by the presence of at least one sialic acid residue in the oligosaccharide moiety. Among gangliosides, α2-3-sialylated glycosphingolipids (α2-3-GSLs) constitute a functionally important subgroup in which a sialic acid residue is linked through an α2–3 glycosidic bond to terminal galactose (Gal) or N-acetylgalactosamine (GalNAc) in the oligosaccharide chain (3). This structural specificity enables selective interactions with cell-surface receptors and lipid-raft components, thereby contributing to the regulation of membrane proteins and ion channels, signal transduction, and intercellular communication (4, 5). As integral components of membrane microdomains, α2-3-GSLs can modulate cellular signaling through their distinctive spatial configurations and charge properties (6, 7), providing a structural and functional basis for examining their roles in pathological networks.

**Figure 1:**
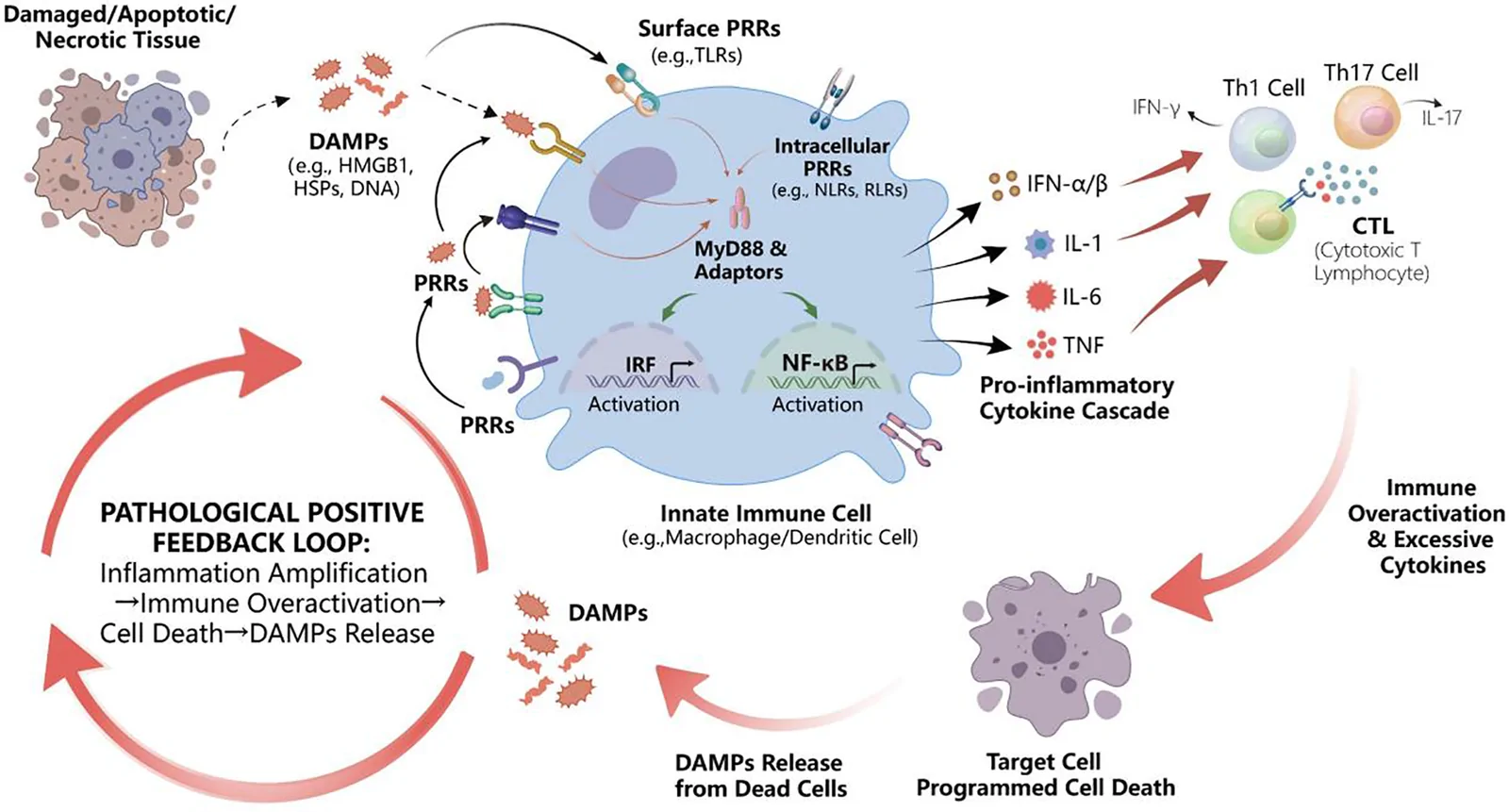
DAMP-associated inflammatory amplification and potential feedback relationships. DAMPs released from injured or dying cells can be recognized by PRRs and, in relevant models, are associated with activation of MyD88-IRF/NF-κB signaling, release of proinflammatory mediators, and recruitment of immune cells. Sustained immune responses may increase PCD in target cells, leading to the release of additional DAMPs. The arrows indicate possible associations inferred from existing studies; their sequence and magnitude are constrained by the experimental model and pathological context.

Maintenance of tissue homeostasis depends on coordinated and dynamically balanced interactions among inflammation, immune responses, and programmed cell death (PCD) (8, 9). Rather than operating independently, these defense processes engage in extensive molecular crosstalk to form an interconnected regulatory network that clears harmful stimuli and preserves internal stability (10, 11). Physiological balance within this network supports normal tissue function, whereas its disruption may contribute to the development and progression of diverse diseases. Damage-associated molecular patterns (DAMPs) released from injured, apoptotic, or necrotic tissues are directly sensed by cell-surface or intracellular pattern-recognition receptors (PRRs) on innate immune cells (12). Through adaptor proteins such as MyD88, PRRs rapidly activate interferon regulatory factor (IRF) and nuclear factor κB (NF-κB) signaling, inducing cascades of proinflammatory cytokines, including interferon-β (IFN-β), interleukin-1β (IL-1β), tumor necrosis factor-α (TNF-α), and interleukin-6 (IL-6), which initiate local inflammation and recruit immune cells (13–15). Excessive immune activation, however, can result in cytokine overproduction, sustained immune-cell activation, tissue injury, and subsequent cell death. Overactivated Th1 and Th17 cells secrete Interferon-gamma (IFN-γ) and Interleukin-17 (IL-17) and, together with cytotoxic T lymphocytes (CTLs), promote PCD in target cells. DAMPs released from these dying cells can further stimulate PRRs (16), potentially amplifying interconnected pathological events in certain diseases or models. The relationships shown in **Figure 1** represent a conceptual synthesis and do not imply that every step has been causally validated in sequence within a single experimental system.

**Figure 2:**
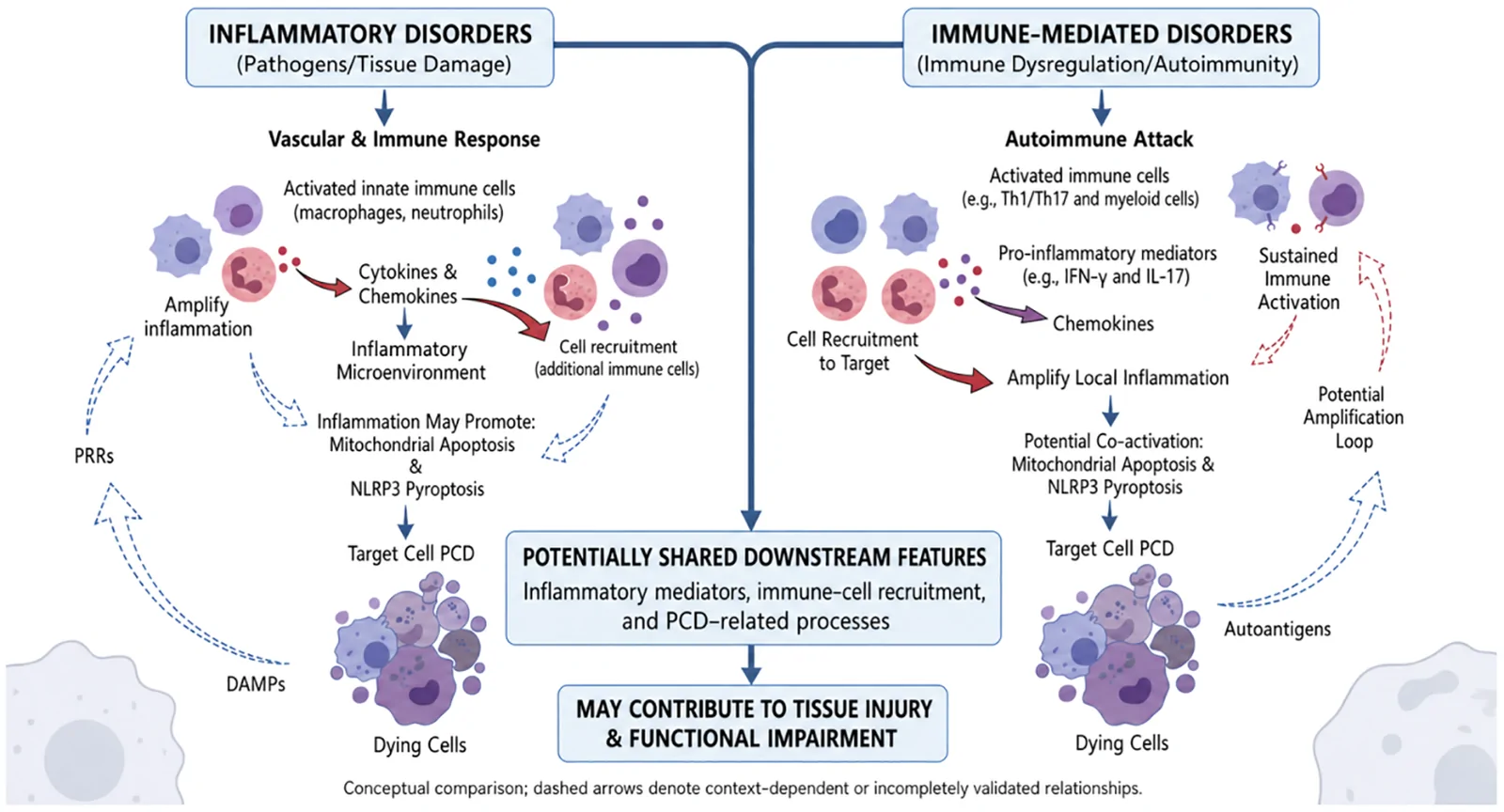
Potentially shared downstream nodes linking inflammation, immune responses, and programmed cell death in inflammatory and immune-mediated diseases. DAMP-PRR-associated innate immune responses (left) and self-antigen-directed immune injury (right) have distinct initiating factors, but both can be accompanied in certain models by production of proinflammatory mediators, immune-cell infiltration, and mitochondrial apoptosis or NLRP3-associated pyroptosis. This figure provides a conceptual comparison and does not imply that the two disease categories share identical pathways or a fixed causal sequence.

Both inflammatory and immune-mediated diseases can involve dysregulation of this network, although their initiating factors and pathway combinations differ (17–19). Inflammatory diseases typically initiate coordinated vascular and innate immune-cell responses to eliminate invading pathogens or damaged cells (20, 21), whereas immune-mediated diseases are characterized by defective immune recognition and inappropriate attack on normal host tissues, often accompanied by selective overproduction of proinflammatory mediators such as IFN-γ and IL-17 (22). Despite these distinct initiating mechanisms, their downstream effects may converge. During disease progression, both categories can upregulate chemokine networks involving Interleukin-8 (IL-8) and C-C motif chemokine ligand 2 (CCL2), thereby recruiting peripheral neutrophils and macrophages to target organs. The combined effects of infiltrating immune cells and local sterile inflammation may activate mitochondrial apoptosis and NOD-like receptor family pyrin domain-containing 3 (NLRP3) inflammasome-associated pyroptosis, ultimately promoting PCD in target cells (16). Moreover, DAMPs and exposed self-antigens released after cellular disintegration can reactivate inflammatory responses through PRRs (17) and sustain immune-cell activation (11). These events may therefore form a mutually reinforcing relationship involving inflammatory or immune activation, PCD of target cells, release of damage-associated substrates, and reactivation of inflammatory and immune networks. Collectively, these processes can be viewed as potentially shared downstream pathological nodes, although their combinations, directions of effect, and supporting evidence vary by disease, disease stage, and experimental model (8) (**Figure 2**).

Although previous studies suggest that α2-3-GSL subtypes have distinct regulatory or pathological roles in inflammatory activation, immune homeostasis, and PCD (23–27), their multitarget coordination, antagonistic mechanisms, and cell-context-dependent functions within the broader network have not been systematically characterized. Most available evidence derives from single experimental systems or specific pathological models. Consequently, current understanding of how lipid-raft remodeling mediates downstream signaling crosstalk remains fragmented, and the depth of evidence varies substantially among subtypes; protective mechanisms are relatively well characterized for GM1, whereas the functions of most other subtypes remain preliminary (28, 29). Moreover, the biological roles of these lipids differ across diseases. In central neurodegenerative disorders, they are studied mainly as homeostatic signaling modulators, whereas in certain peripheral autoimmune diseases, such as Guillain–Barré syndrome, they can serve as pathogenic targets of immune attack (4, 30).

Accordingly, this review uses representative neurological diseases with distinct mechanisms as analytical settings to systematically assess spatiotemporal changes in α2-3-GSL profiles and their associations with inflammation, immune responses, and PCD. Based on the available evidence, we compare the potentially shared downstream regulatory nodes involving α2-3-GSLs across pathological contexts, while also emphasizing disease-specific features. We further examine their possible links to DAMP recognition, death-receptor assembly, and related signaling crosstalk. Rather than assuming that α2-3-GSLs follow a uniform mechanism across diseases, we propose a conceptual lipid–inflammation–immunity–death framework that requires further validation and may guide subsequent disease-specific functional studies and precision interventions while accounting for both shared and divergent features.

## 2 Regulatory roles and disease relevance of α2-3-GSLs in neuroinflammation, immune responses, and programmed cell death

This section focuses on neurological diseases, including neurodegenerative disorders such as Parkinson’s disease (PD), Alzheimer’s disease (AD), and Huntington’s disease (HD), as well as neuroimmune disorders such as multiple sclerosis (MS) and Guillain–Barré syndrome (GBS). We review how interactions among inflammation, immune dysregulation, and PCD may contribute to disease progression. Focusing on major subtypes such as GM1, GD1a, and GD3, we examine how these lipids may influence these pathological processes through Toll-like receptor 4 (TLR4)/NF-κB, complement, and related pathways; modulate T- and B-cell function; shape cell-death programs; and support neural homeostasis in distinct neuropathological microenvironments.

The neurodegenerative diseases considered here are characterized by the aggregation of proteins such as α-synuclein (α-Syn), amyloid-β (Aβ), and mutant huntingtin (mHTT), whereas immune-mediated diseases primarily involve dysregulated autoimmune recognition. Despite these distinct initiating factors, α2-3-GSLs may act at multiple levels of inflammation, immune responses, and cell death. By disentangling these subtype-specific behaviors and pathway interactions, this section establishes a basis for identifying shared and disease-specific features of the proposed lipid–inflammation–immunity–death regulatory framework.

### 2.1 Parkinson’s disease: regulation of α-syn-associated neuroinflammation, immune responses, and programmed cell death

PD is a chronic neurodegenerative disorder characterized by progressive loss of dopaminergic neurons in the substantia nigra and abnormal accumulation of α-Syn. Growing evidence suggests that dysregulated interactions among neuroinflammation, immune activation, and PCD contribute to PD progression (30, 31). Persistent aberrant microglial activation is considered an important component of this process (32). In this context, α2-3-GSLs, particularly GM1, GD1a, and GD3, may modulate several pathological events through subtype-specific direct or indirect associations with α-Syn and related pathways (28, 33, 34).

**Figure 3:**
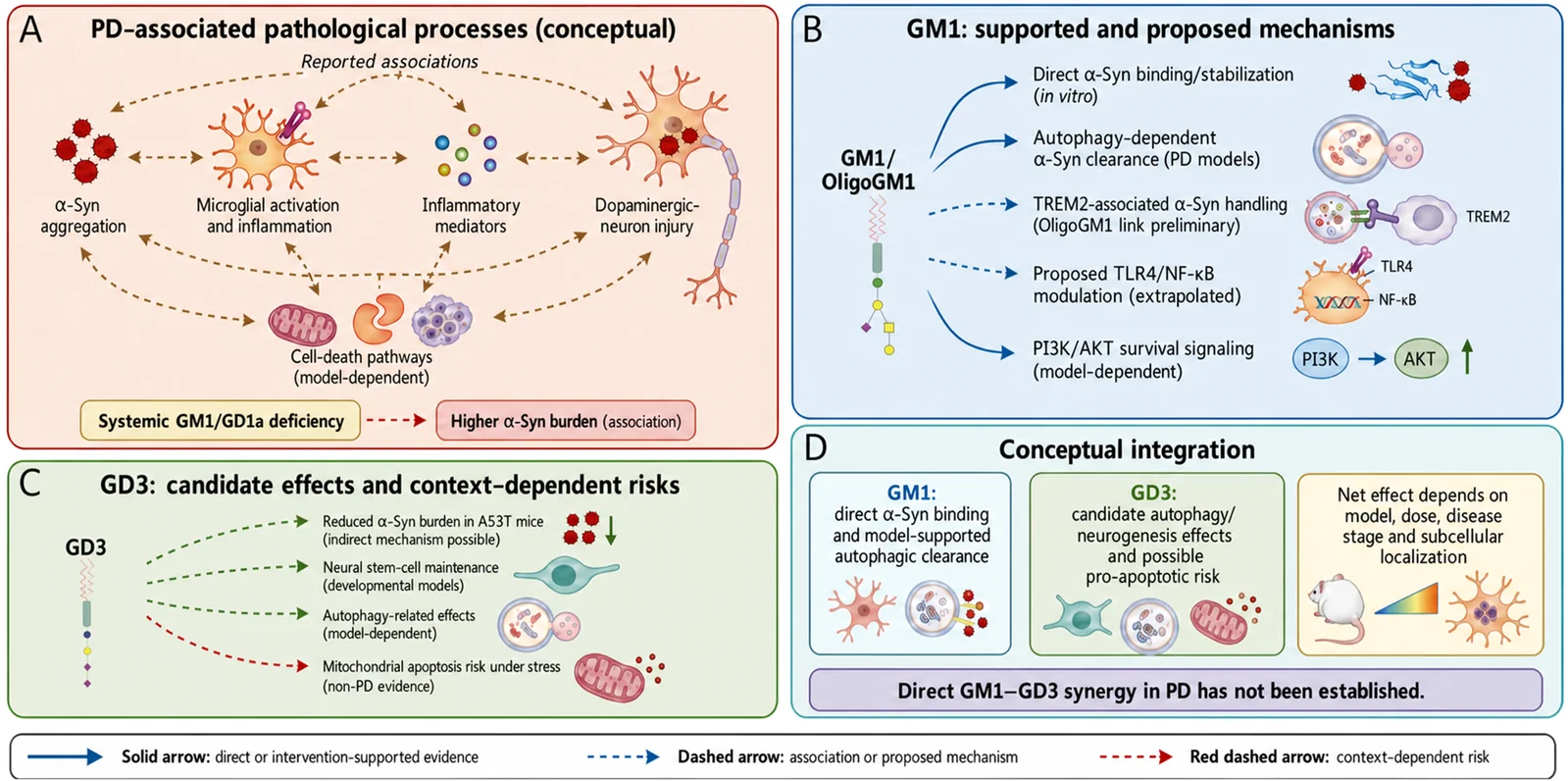
Evidence-integrated schematic of GM1/GD1a- and GD3-associated processes involving α-Syn, inflammation, autophagy, and cell death in PD. **(A)** associations among reduced GM1/GD1a levels, α-Syn aggregation, microglial activation, and neuronal injury. **(B)** GM1/OligoGM1 stabilize α-Syn conformation in biochemical systems and selected disease models and promote autophagic clearance in specific PD models. TREM2 may contribute to α-Syn uptake and processing; however, a direct link among OligoGM1, TREM2 dependence, and autophagic degradation has not been established, and the lipid raft–TLR4 mechanism remains an extrapolation from other model systems. **(C)** GD3-associated effects on neurogenesis, autophagy regulation, and cell survival are supported primarily by developmental models, general cellular systems, and limited PD models and may be constrained by its proapoptotic activity. **(D)** conceptual integration of the potential effects of individual subtypes; this panel does not represent a validated synergistic causal pathway.

Systemic reductions in GM1 and GD1a have been reported in patients with PD, not only in central tissues such as the substantia nigra but also in peripheral tissues, including the colon and skin. These findings suggest that disrupted GSL metabolism may be a susceptibility factor for sporadic PD. Reduced GM1 and GD1a levels are associated with increased abnormal α-Syn aggregation. Aggregated α-Syn can act as both an endogenous DAMP and a pathological seed, activating microglial inflammatory responses and potentially increasing neuronal injury and the protein-aggregation burden (**Figure 3A**) (35).

#### 2.1.1 Multidimensional network regulation by GM1

Suppressing α-Syn aggregation and coupling immune clearance to autophagy: Abnormal α-Syn aggregation can activate microglia and initiate neuroinflammation. Aggregated α-Syn induces microglial release of proinflammatory mediators, including TNF-α and IL-1β, and reciprocal interactions among α-Syn pathology, immune activation, and neuroinflammation may contribute to the progressive loss of dopaminergic neurons (36, 37). Studies of GM1-mediated regulation of α-Syn have examined both the protective effects of the intact molecule *in vivo* and the functional contribution of its oligosaccharide moiety. At the whole-molecule level, *in vitro* and *in vivo* evidence supports a role for GM1 in maintaining α-Syn homeostasis. Systemic depletion of endogenous GM1 is a pathological feature associated with PD progression, and GM1 may exert neuroprotective effects by stabilizing α-Syn in a nonaggregated, functional state (38). At the molecular level, GM1 directly binds α-Syn and stabilizes its α-helical conformation, thereby inhibiting conversion to toxic fibrillar forms (39). Animal studies provide further support: GM1-deficient mice develop motor impairment associated with abnormal α-Syn aggregation in the substantia nigra, whereas treatment with blood-brain barrier-permeable GM1 analogues or subcutaneous GM1 has reduced α-Syn pathology and improved neuronal integrity in relevant models (40–44). At the level of the functional domain, the GM1 ganglioside oligosaccharide (OligoGM1) has been identified as a key active structure underlying these neuroprotective effects. Cell-free and *in vitro* reconstituted systems show that isolated OligoGM1 can directly bind α-Syn monomers and inhibit abnormal aggregation (28, 45). Preliminary evidence suggests that, in addition to directly inhibiting extracellular α-Syn aggregation, OligoGM1 may modulate microglial activation. In an HMC3 human microglial model, OligoGM1 pretreatment reduced the intracellular α-Syn burden in TREM2-positive cells following exposure to α-Syn pre-formed fibrils (PFFs) and decreased activation-related measures, including Iba1 expression and IL-6 release (45). An independent study showed that TREM2 can directly bind PFFs and facilitate their uptake by microglia; however, lysosomal proteolysis of internalized fibrils may generate fragments with greater seeding activity, indicating that uptake does not necessarily equate to effective clearance (46) Because the OligoGM1 study did not include TREM2 blockade or genetic deletion and did not measure autophagic flux, direct OligoGM1 dependence on TREM2 and its linkage to autophagic degradation remain preliminary.

GM1-mediated regulation of α-Syn pathology through autophagy is supported by relatively extensive *in vitro* and *in vivo* evidence. In a subacute 1-methyl-4-phenyl-1,2,3,6-tetrahydropyridine (MPTP) mouse model of PD and a cellular injury model induced by 1-methyl-4-phenylpyridinium ion (MPP^+^), GM1 increased phosphorylation of the upstream autophagy proteins Unc-51-like kinase 1 (ULK1) and autophagy-related protein 13 (ATG13), activated autophagic flux, reduced intracellular α-Syn accumulation, improved mitochondrial function, and reduced oxidative stress. Pharmacological inhibition of autophagy abolished the neuroprotective effect of GM1, providing causal evidence for this pathway within these models (47). By promoting intracellular degradation, autophagy activation may reduce the release of toxic proteins as DAMPs into the local microenvironment and thereby attenuate subsequent neuroinflammatory signaling at an early stage of the pathological process (33, 47, 48).

Direct modulation of inflammatory and immune pathways: TLR4 is a major innate immune-recognition receptor on microglia. Its activation initiates NF-κB signaling and induces the release of proinflammatory mediators (49, 50). Studies in BV2 cells, mice, rats, and primary human microglia have reported that GM1 suppresses excessive lipopolysaccharide-induced NF-κB activation and reduces the secretion of IL-1β, TNF-α, and IL-6 (26, 51). The proposed upstream mechanism is commonly framed as the lipid-raft regulatory hypothesis. In PC12 cells and primary epithelial cells, GM1 and GD1a have been shown to alter membrane lipid-raft organization, prevent TLR4 translocation into raft domains and assembly of signaling complexes, and thereby suppress downstream inflammatory signaling (52). This mechanism has been extrapolated to microglia but has not yet been directly validated in microglial systems (**Figure 3B**).

#### 2.1.2 Candidate regulatory and neurogenic effects of GD3

GD3, an α2-3-GSL highly expressed on neural stem cells, has potential roles in neural repair, proteostasis, and inflammatory regulation in PD. However, most of these effects have been inferred from general cellular systems and developmental models. Its actual role in the pathological microenvironment of chronic roteinopathy in PD therefore requires dedicated *in vivo* validation in the nigrostriatal pathway.

Regulation of neurogenesis: established developmental functions and limitations of extrapolation to PD. GD3 is an α2-3-GSL marker of neural stem and progenitor cells, and its roles in supporting self-renewal, maintaining stemness, and promoting neuronal differentiation are well established in developmental neurobiology. Genetic studies have shown that deficiency of GD3 synthase results in progressive impairment of endogenous adult neurogenesis in the mouse hippocampus and other brain regions (53, 54). This effect is largely mediated by interactions between GD3 and the epidermal growth factor receptor (EGFR), which stabilize receptor signaling (55). Although this mechanism provides a rationale for endogenous regeneration of dopaminergic neurons in PD, the supporting evidence is derived primarily from *in vitro* stem-cell cultures and developmental studies in healthy animals. The pathological microenvironment of PD, characterized by chronic neuroinflammation, abnormal α-Syn deposition, and high oxidative stress, may markedly impair neural stem-cell survival and proliferation and alter lineage specification. To date, GD3 has been shown to improve neurogenesis only in the olfactory bulb of A53T α-Syn-transgenic mice; evidence that it induces functional dopaminergic neurogenesis and circuit integration in the nigrostriatal pathway remains insufficient (44).

α-Syn pathology and neuroinflammatory signaling: Direct biochemical evidence that GD3 physically regulates α-Syn aggregation is currently lacking. In A53T α-Syn-transgenic mouse models of PD, intranasal GD3 administration was associated with reduced cerebral α-Syn accumulation, an effect that may involve indirect processes such as autophagic clearance and neurogenesis (34). Because aggregated α-Syn can act as a DAMP and activate microglial TLR4/NF-κB signaling, GD3-associated effects could theoretically influence the interconnected processes of α-Syn deposition, glial activation, and neuronal injury; however, this proposed pathway has not been directly validated in PD models. The abundance of other gangliosides and the functional state of the autophagy-lysosome system may also modify the net effect. Moreover, GD3 can translocate to mitochondria and promote apoptosis under acute stress, and its long-term *in vivo* safety remains unclear (56).

Regulation of autophagic flux and model boundaries: GD3 can localize to autophagic membranes and colocalize with microtubule-associated protein 1 light chain 3 (LC3), phosphatidylinositol 3-phosphate (PI3P), and lysosome-associated membrane protein 1 (LAMP1). In human fibroblasts, knockdown of GD3 synthase directly blocked autophagic flux, whereas exogenous GD3 supplementation reversed this effect (25). Within lipid-raft-like structures of mitochondria-associated membranes (MAMs), GD3 may also participate in functional interactions involving autophagy and Beclin 1 regulator 1 (AMBRA1) and the endoplasmic-reticulum chaperone calnexin (57). In cellular models of PD, broad inhibition of ganglioside synthesis has been associated with impaired autophagy, α-Syn accumulation, and cell death, whereas GD3 repletion partially restored autophagic flux and cell survival (58). These findings support a role for GD3 in autophagy regulation, although its disease-specific effects require *in vivo* validation.

Although GD3 could theoretically facilitate autophagosome maturation by serving as a lipid-raft scaffold within MAM domains (57), this regulatory axis may fail in the context of chronic proteinopathy in PD, and the proposed mechanism currently lacks direct support from PD models. On the one hand, degenerating neurons in PD commonly exhibit impaired lysosomal acidification and severe α-Syn overload, creating a substrate-clearance bottleneck within the autophagy-lysosome pathway. Disrupted GSL metabolism and lysosomal dysfunction can form a mutually reinforcing cycle (59). Against this background, GD3 may be retained within dysfunctional autolysosomal compartments as autophagic flux stalls, potentially worsening substrate congestion. On the other hand, GD3 function at MAMs is closely coupled to mitochondrial dynamics. Under cluster of differentiation 95/factor associated suicide (CD95/Fas) death-receptor signaling, GD3 accumulates at mitochondrial fission sites and, together with human fission 1 (hFis1) and related fission proteins, forms a multiprotein apoptotic signaling complex that promotes excessive mitochondrial fission and opening of the mitochondrial permeability transition pore (60). Neurobiological studies have further shown that GD3 directly binds dynamin-related protein 1 (Drp1) and regulates its turnover and mitochondrial fission activity (61). Because mitochondrial-network homeostasis is already disrupted in PD neurons, pathological GD3 accumulation could shift physiological autophagy initiation toward excessive mitochondrial fission and ultimately reduce the overall efficiency of autophagic clearance (**Figures 3C, D**). **Figure 3D** presents a conceptual integration; direct synergy between GM1 and GD3 in PD has not been established, and their net effects may depend on the model, dose, disease stage, and subcellular localization.

### 2.2 Alzheimer’s disease: bidirectional regulation of the Aβ metabolism-associated inflammation–immunity–autophagy network

Alterations in brain GSLs in AD are spatially heterogeneous: although overall GM1 and GD1a levels are reduced across the brain, GM1 ganglioside-bound amyloid β-protein (GAβ) complexes may be locally enriched within lipid-raft microdomains (62).

**Figure 4:**
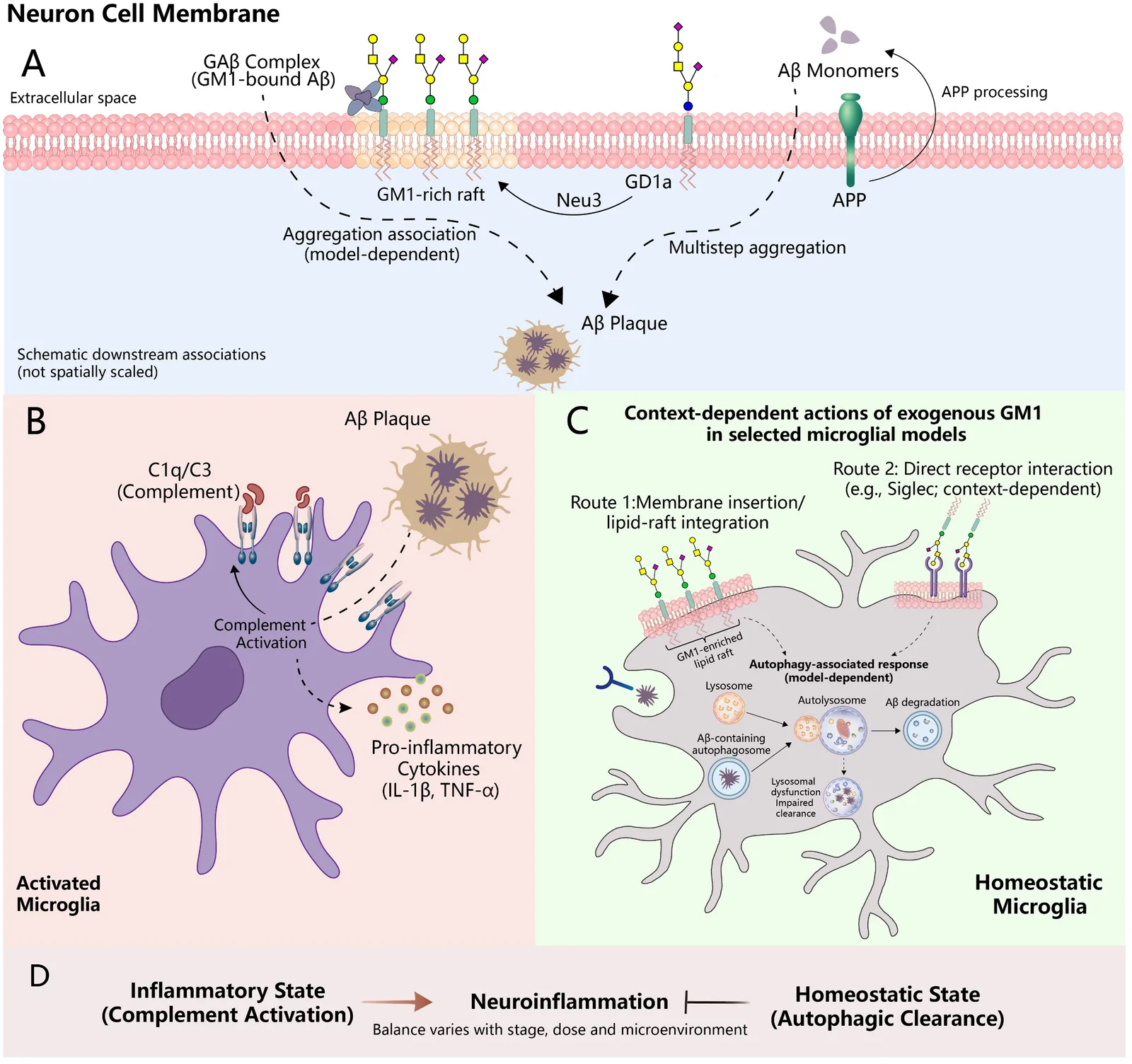
Spatial abnormalities in GM1/GD1a distribution and processes related to Aβ, complement, and autophagy in AD. **(A)** APP processing generates Aβ monomers that undergo multistep aggregation. Within GM1-enriched lipid rafts, GM1-bound Aβ (GAβ) may facilitate aggregation in a model-dependent manner, while Neu3-mediated desialylation of GD1a generates GM1 and may alter local membrane composition. Downstream associations are schematic and not spatially scaled. **(B)** Aβ plaque-associated C1q/C3 complement activation is linked to microglial activation and the release of pro-inflammatory cytokines, including IL-1β and TNF-α. **(C)** In selected microglial models, exogenous GM1 may act through membrane/lipid-raft incorporation or context-dependent receptor interactions, such as those involving Siglecs, to support autophagy-associated Aβ processing. Lysosomal dysfunction may nevertheless impair autolysosomal clearance. **(D)** The balance between complement-associated neuroinflammation and autophagic clearance varies with disease stage, GM1 dose, and the local microenvironment.

α2-3-GSLs, predominantly GM1 and GD1a, may participate in the compartmentalized processing of amyloid precursor protein (APP) and the transmembrane metabolism of Aβ (**Figure 4A**). Autopsy studies have shown overall reductions in GM1 and GD1a in AD-affected brain regions, particularly in patients with early-onset disease (63); concurrently, GAβ may be locally enriched within lipid-raft microdomains. GM1 in these microdomains may promote Aβ conformational changes and protofibril formation, although its causal contribution to AD onset and progression requires further validation (64, 65). The current evidence has three major limitations. First, GSL abnormalities may either contribute to the disease process or arise secondarily from neuronal loss. Second, the net effect of GM1 depends on dose, disease stage, and the membrane microenvironment. Third, human data are derived mainly from end-stage postmortem tissue, and *in vivo* longitudinal and pathway-specific validation remains lacking (66).

#### 2.2.1 Bidirectional homeostatic regulation of the inflammation–immunity–autophagy network by GM1

Aβ-associated immune and inflammatory responses: GAβ complexes and higher-order Aβ aggregates may act as endogenous DAMPs, be recognized by pattern-recognition receptors on resident innate immune cells in the central nervous system, and promote activation of the classical complement pathway (67). Complement components such as C1q and C3 accumulate around amyloid plaques; this accumulation may be accompanied by proinflammatory microglial responses and the release of IL-1β, TNF-α, IL-6, and other mediators. Sustained complement activation may also contribute to aberrant pruning of cholinergic synapses and impaired neural-network function (68, 69) (**Figure 4B**).

The effects of complement are stage dependent. Early in the disease, opsonization by C1q and C3 may facilitate microglial phagocytosis and clearance of Aβ. During chronic progression, however, sustained complement activation may increase the risk of aberrant synaptic pruning, synapse loss, and cognitive decline (70–72).

GSL homeostasis may help maintain neuroimmune balance. Mice lacking both GM2/GD2 synthase and GD3 synthase (double knockout, DKO) exhibit complement activation, microglial proliferation, and neurodegenerative phenotypes even in the absence of exogenous Aβ stimulation (73). Together with evidence of local GAβ enrichment, these findings suggest that both focal overaccumulation and widespread GSL deficiency may disrupt immune homeostasis. GSL deficiency may also alter lipid-raft architecture, weaken endogenous glycan-dependent inhibitory signaling through sialic acid-binding immunoglobulin-like lectins (Siglecs) such as CD33, and thereby promote disinhibited microglial activation (74). Thus, the net effects of α2-3-GSLs depend on their abundance and distribution within membrane microdomains (**Figure 4B**). Two membrane-associated actions of exogenous GM1 should be distinguished. Exogenous GM1 can spontaneously insert into the outer leaflet of the plasma membrane and partition into lipid rafts, thereby influencing the membrane organization, clustering, or trafficking of pattern-recognition receptors such as TLR4. Depending on the cellular and species context, its exposed glycan may also interact directly with selected surface receptors, including certain Siglecs (52, 74–76). These routes are not mutually exclusive, and their relative contributions to downstream autophagy–lysosome responses remain model dependent (**Figure 4C**).

Autophagy-associated protection and its effects on immune homeostasis: Autophagy contributes to the lysosomal degradation of Aβ aggregates. In selected models, GM1 enhances autophagic flux by inhibiting microglial protein kinase B (AKT)/mechanistic target of rapamycin (mTOR) signaling, thereby promoting Aβ degradation and reducing activation of the NLRP3 inflammasome (77). Studies of microglia-targeted, autophagy-modulating nanotherapeutics have further shown that restoration of autophagy–lysosome function can improve Aβ clearance, reduce complement C3 activation, and attenuate neuroinflammation (78). These findings suggest that GM1-associated autophagic regulation may link Aβ clearance to attenuation of inflammation, although its therapeutic effects require further validation (**Figures 4C, D**). Notably, the GM1–autophagy–inflammation findings described above are reported in a recent preprint by Wang et al. (2025), which has not yet undergone peer review and should therefore be interpreted as preliminary, model-level evidence (77).

These effects are dose and disease-stage dependent. Moderate enhancement of autophagy may promote Aβ degradation, whereas sustained overactivation may increase the risk of autophagic death in microglia (79). Chronically affected AD brain regions frequently exhibit reduced vacuolar-type H^+^-ATPase (V-ATPase) activity and insufficient lysosomal acidification, which may limit the degradation of autophagic substrates (80, 81). If upstream autophagosome formation is increased without restoring downstream lysosomal function, undegraded vesicles may accumulate further. Moreover, the relationship between autophagy and complement is not unidirectionally inhibitory; its net effect also depends on disease stage and the local microenvironment (68) (**Figures 4C, D**).

#### 2.2.2 Metabolic conversion and potential cooperative effects of GD1a: evidentiary boundaries

GD1a is a major α2-3-GSL subtype and decreases in parallel with GM1 in AD-affected brain regions (63). GD1a is structurally similar to GM1 and can be converted into GM1 through removal of its terminal sialic acid by membrane-bound neuraminidase 3 (Neu3); it may therefore serve as a metabolic source of GM1. Whether this conversion subsequently affects APP processing and the transmembrane metabolism of Aβ requires direct validation (82, 83) (**Figure 4A**). GD1a may also act with GM1 in maintaining lipid-raft stability and modulating Aβ oligomerization and microglial inflammatory responses, although the available evidence is based largely on structural analogy and extrapolation across models (26, 84, 85) (**Figure 4A**).

The proposed GD1a-related mechanisms remain subject to substantial evidentiary limitations. Most conclusions are based on its structural similarity to GM1, with little subtype-specific *in vivo* validation in AD animal models (84). Neu3 expression and activity may also change with disease stage, showing a compensatory increase early but declining as neuronal degeneration advances (86–88). Because GD1a can be dynamically converted into GM1, its effects on lipid rafts and microglia cannot be fully distinguished from those of GM1, its conversion product (27, 66). Consequently, current studies cannot exclude the possibility that some of the observed protective effects are mediated primarily by GM1 (89). The independent functions of GD1a and its feasibility as a therapeutic target require further investigation.

### 2.3 Huntington’s disease: mHTT-associated regulation of inflammation, apoptosis, and autophagy

**Figure 5:**
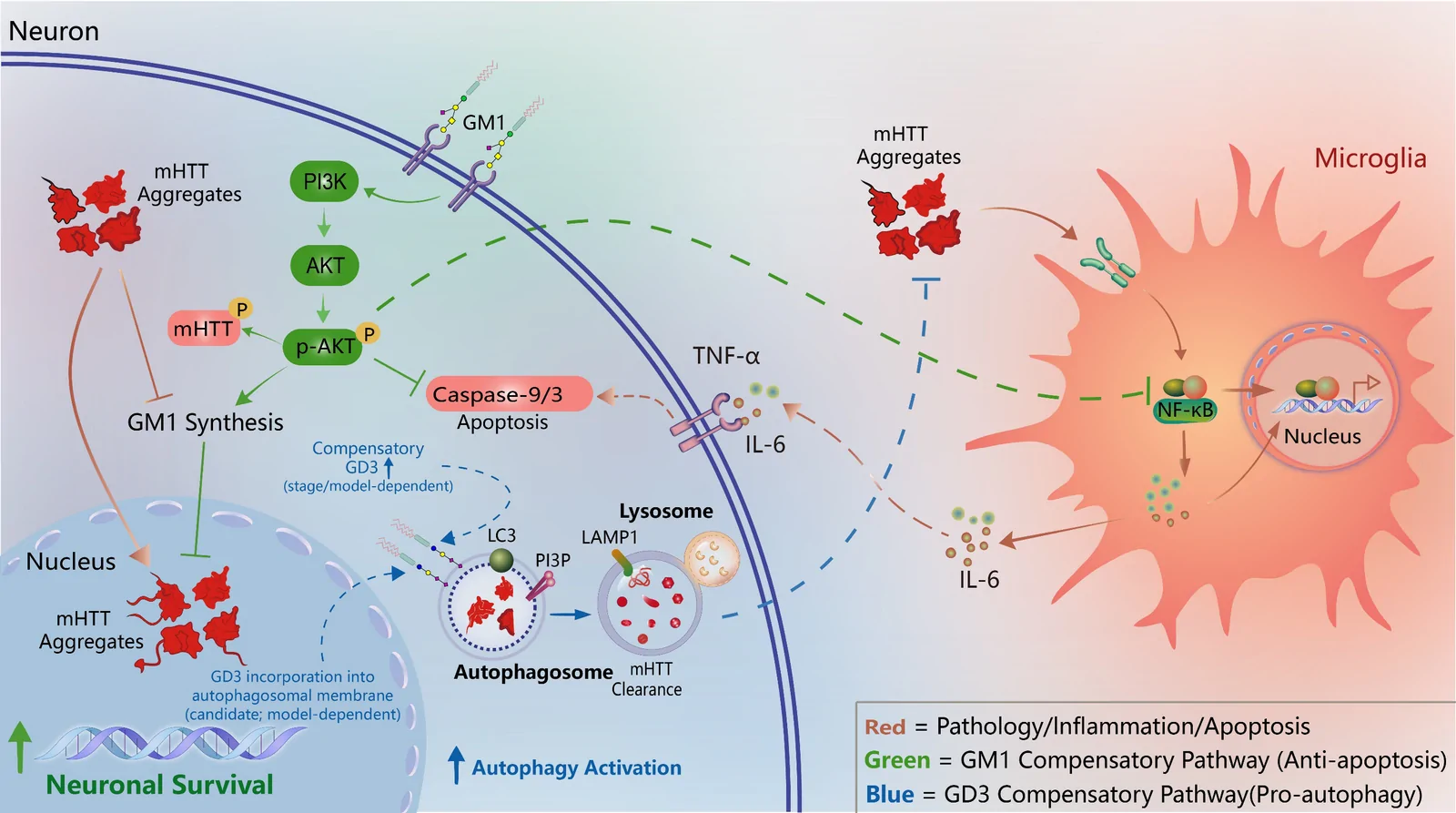
mHTT-associated abnormalities in inflammation, apoptosis, and autophagy in HD and the candidate regulatory effects of GM1/GD3. The red pathway summarizes potential reciprocal interactions among mHTT aggregation, GSL abnormalities, inflammation, and impaired clearance. The green pathway indicates that GM1 is associated with PI3K/AKT-related survival signaling, attenuation of apoptosis, and inflammatory modulation in selected models. The blue pathway depicts GD3 as an autophagosomal membrane-associated lipid potentially involved in LC3/PI3P-related autophagy and mHTT clearance, rather than acting through an undefined autophagosomal membrane receptor. These effects are model-, dose-, and disease-stage-dependent.

HD is an autosomal dominant neurodegenerative disorder characterized primarily by mHTT aggregation and injury to striatal medium spiny neurons. Neuroinflammation, apoptosis, and autophagic defects may influence one another, and metabolic alterations in α2-3-GSLs are associated with these processes (84). Both autopsy and model studies have reported reductions in GM1 and GD1a accompanied by increased GD3 in HD-affected brain regions (90). mHTT may also suppress the expression or activity of enzymes involved in ganglioside biosynthesis, thereby reducing GM1 production (91). GSL dysregulation and impaired mHTT clearance may reinforce each other (**Figure 5**, red pathway), whereas GM1 and GD3 may exert compensatory effects through survival signaling and autophagy-related processes, respectively (25, 92–94).

#### 2.3.1 GM1-mediated regulation: PI3K/AKT activation and attenuation of inflammation and apoptosis

The protective effects of GM1 primarily involve the inhibition of apoptosis and modulation of inflammation and may be mediated through pro-survival phosphatidylinositol 3-kinase/protein kinase B (PI3K/AKT) signaling.

Evidence related to antiapoptotic effects: Studies in cultured HD striatal cells, patient-derived fibroblasts, and several transgenic mouse models of HD have shown that exogenous GM1 can reduce mHTT-associated cytotoxicity, improve cell survival, and ameliorate motor and cognitive phenotypes in some models (28, 91). These studies suggest that GM1 may increase the p-AKT/AKT ratio through lipid raft-associated PI3K/AKT signaling and partially restore AKT signaling in HD models (91). Activated AKT can promote phosphorylation of the mHTT N terminus at Ser13/Ser16, thereby reducing toxic cleavage fragments and intranuclear aggregation. This finding is consistent with evidence that insulin-like growth factor 2 (IGF2)-dependent AKT signaling promotes mHTT clearance (95, 96). PI3K/AKT activation may also inhibit apoptosis-related proteins, including cysteine-aspartic acid protease 3 (caspase-3) and cysteine-aspartic acid protease 9 (caspase-9), thereby helping to attenuate mitochondria-dependent apoptosis associated with mHTT accumulation (91) (**Figure 5**, green pathway).

Inflammation-related effects: mHTT aggregation can disrupt the central nervous system microenvironment and promote microglial activation and the release of proinflammatory mediators such as IL-6 and TNF-α. Chronic inflammation may in turn sensitize neurons to apoptotic signals. By modulating PI3K/AKT and NF-κB signaling, GM1 may affect both apoptosis and inflammation and thereby limit their reciprocal amplification (92, 97) (**Figure 5**, green pathway).

GM1-associated protective effects are constrained by the model, dose, and disease stage. Current conclusions derive primarily from *in vitro* experiments, short-term pretreatment paradigms, and local administration in young HD mice; whether these effects persist at advanced disease stages remains unclear. Late-stage disruption of lipid rafts and GSL biosynthetic pathways may also reduce the membrane localization and signaling capacity of exogenous GM1 (91). In addition, sustained overactivation of PI3K/AKT/mTOR may inhibit autophagy initiation; GM1 may therefore have nonlinear dose- and time-dependent effects (84). Its anti-inflammatory effects have been validated mainly in simplified culture systems and remain to be confirmed in neuron–microglia cocultures or chronic inflammatory settings (97). The long-term effects of GM1 supplementation on endogenous GSL homeostasis and wild-type HTT function have not been systematically evaluated (29).

#### 2.3.2 Compensatory regulation by GD3: autophagy-related clearance of toxic proteins

Increased GD3 in the HD brain may represent a compensatory form of GSL metabolic remodeling and may influence mHTT clearance through autophagy regulation. These effects could complement GM1-associated modulation of apoptosis and inflammation.

Autophagic dysfunction and mHTT aggregation may reinforce each other in HD: mHTT deposition may further impair autophagosome–lysosome fusion and protein clearance (98–100). Available studies suggest that GD3 may participate in autophagy-related processes and facilitate mHTT clearance (101). Whether this change subsequently attenuates microglial activation and neuronal apoptosis requires disease-specific experimental validation (**Figure 5**, blue pathway). GD3-associated autophagic regulation is therefore more appropriately regarded as a candidate compensatory mechanism than as an established sequential protective pathway.

GD3-associated effects also depend on disease stage and the cellular environment. A modest increase in GD3 early in HD may be associated with improved autophagic flux. During the middle and late stages, however, continued mHTT accumulation can disrupt the cytoskeleton, vesicular sorting, and autophagosome–lysosome fusion; GD3 accumulation may then coincide with autophagosome buildup and increased metabolic stress (102–104). Current evidence is derived primarily from *in vitro* or simplified models, and subtype-specific knockout and rescue studies in transgenic HD animals are lacking. At high levels, GD3 may also participate in proinflammatory signaling in microglia, with its net effect determined by the pathological microenvironment (105, 106). Whether GD3 is directly or indirectly related to mHTT, and through which downstream mechanisms, remains to be determined.

### 2.4 Multiple sclerosis: regulation of inflammation, immune responses, and programmed cell death and their roles in repair

MS is characterized by both autoimmune attack and neurodegeneration. α2-3-GSLs have dual roles in this context: they contribute to myelin homeostasis but may also serve as target antigens for autoantibodies (107, 108). The abundance of α2-3-GSLs, including GM1 and GD1a, is reduced in brain and spinal cord lesions from patients with MS, accompanied by disrupted distribution of myelin membrane lipid rafts, suggesting that these lipids may be involved in central nervous system inflammation and myelin damage (108).

**Figure 6:**
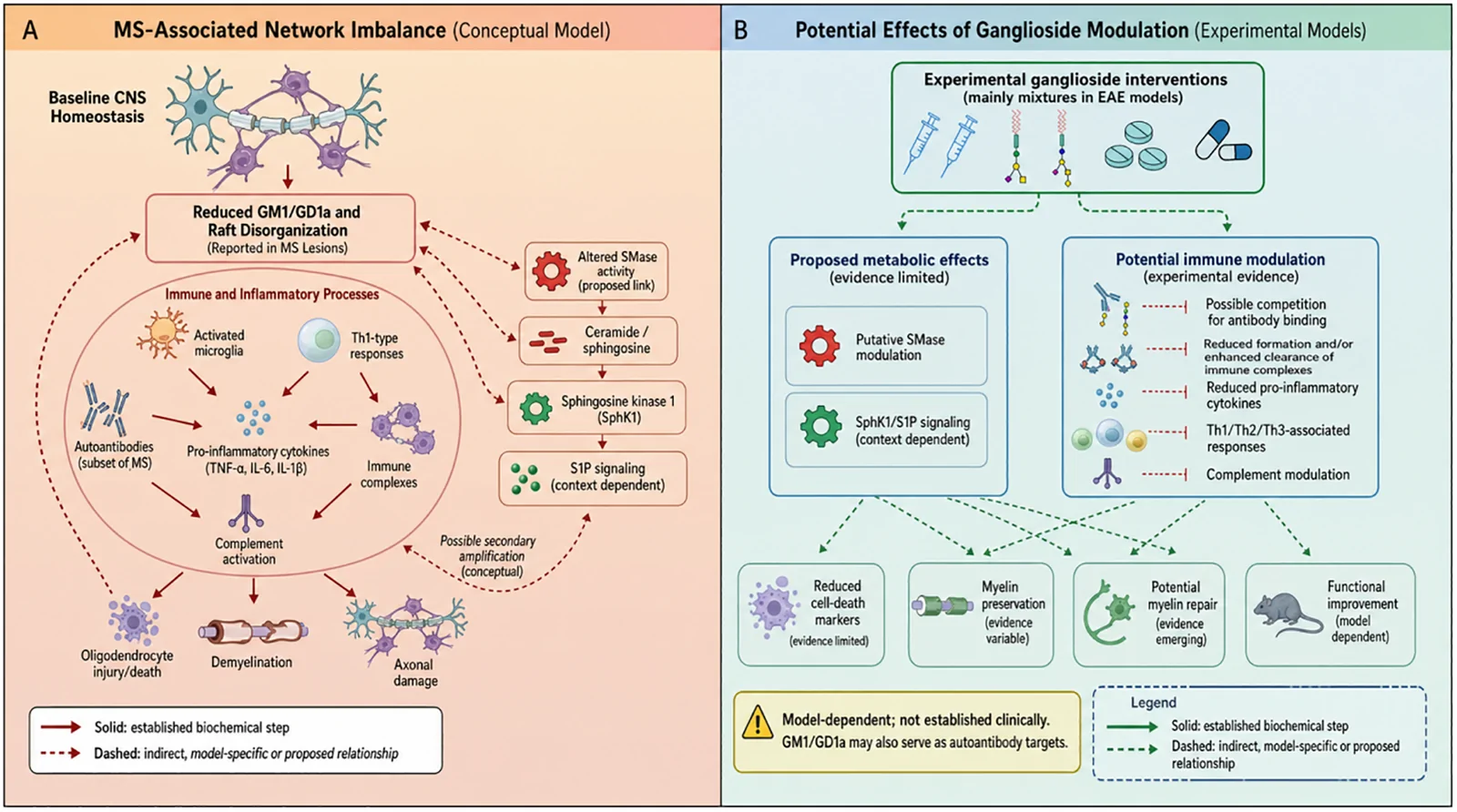
α2-3-GSL-related myelin homeostasis, immune responses, and sphingolipid metabolism in MS. **(A)** GM1/GD1a contribute to MAG-associated axon–myelin interactions and membrane-microdomain homeostasis; their reduction may accompany inflammation, oligodendrocyte injury, and demyelination. These glycolipids may also serve as autoantibody targets. **(B)** EAE and other models associate exogenous glycosphingolipids with immune polarization, regulation of complement/immune complexes, SMase–SphK1/S1P-related metabolic changes, and myelin repair. However, most proposed mechanisms lack disease-subtype-specific and long-term *in vivo* validation, and the figure does not indicate established clinical efficacy.

**Figure 6A** summarizes the potential associations of α2-3-GSLs with axon–myelin interactions, membrane-microdomain homeostasis, immune responses, and sphingolipid metabolism. Evidence for the individual steps is derived from different models and does not establish a single continuous causal pathway. Through interactions with myelin-associated glycoprotein (MAG), α2-3-GSLs may help maintain axon–myelin interactions and lipid-raft homeostasis (108, 109). These lipids have also been linked to complement regulation, T-cell subset balance (23, 26), and sphingomyelinase/sphingosine kinase 1 (SMase/SphK1)-related sphingolipid metabolism (107, 108, 110). Reduced GM1 and GD1a levels may weaken these regulatory effects and are associated with disruption of the SphK1/sphingosine-1-phosphate (S1P) axis, increased inflammation, and oligodendrocyte injury; loss of myelin lipids may, in turn, further exacerbate glycosphingolipid abnormalities (108, 110). These mechanisms are discussed separately in Sections 2.4.1–2.4.3.

#### 2.4.1 Regulation of upstream immune responses

Early MS pathology involves aberrant immune recognition of myelin autoantigens. **Figure 6B** summarizes the candidate regulatory effects of exogenous α2-3-GSLs in experimental models such as experimental autoimmune encephalomyelitis (EAE); it does not imply an established clinical benefit. In EAE, a brain-derived α2-3-GSL mixture containing GM1 and GD1a reduced Th1 cytokine expression and increased Th2/Th3 cytokine secretion (111). Cronassial, a preparation containing GD1a, GM1, GT1b, and GD1b, has also been associated with attenuated EAE symptoms and delayed neurological deficits (108). Some antimyelin antibodies and circulating immune complexes (CICs) in patients with MS may activate complement and promote inflammatory-cell recruitment (108). Exogenous α2-3-GSLs may attenuate this process by competing for antibody binding and facilitating CIC clearance, although the evidence is derived primarily from experimental models (108). In addition, α2-3-GSL treatment has been associated with reduced lesional levels of TNF-α, IL-6, and IL-1β, which may limit inflammatory-cell recruitment and downstream disruption of sphingolipid metabolism (106).

#### 2.4.2 Regulation of the sphingomyelinase–sphingosine pathway

By influencing sphingolipid metabolism, α2-3-GSLs may regulate the balance between inflammation-associated lipid mediators and repair-related signaling. Here, this process is summarized as regulation of metabolic homeostasis (**Figure 6B**).

Structurally, α2-3-GSLs and sphingomyelin share a common ceramide backbone (112, 113). On this basis, exogenous α2-3-GSLs have been proposed to alter SMase substrate recognition and catalytic activity through competitive binding or allosteric modulation, thereby reducing the production of pro-inflammatory lipids such as ceramide and sphingosine. However, current evidence is derived largely from studies of mixed ganglioside preparations, and direct subtype-specific validation remains lacking (108).

Downstream, the pro-inflammatory cytokines TNF-α and IL-6 can enhance SphK1 expression and activation through transcriptional and post-translational mechanisms, increasing pathological S1P production and amplifying inflammatory-cell infiltration and signaling (114, 115). Accordingly, the anti-inflammatory effects of α2-3-GSLs may be accompanied by reduced SphK1 activity and pathological S1P production (114, 116). The resulting improvement in the inflammatory microenvironment may favor oligodendrocyte progenitor-cell proliferation and differentiation and promote remyelination. Preliminary support for this possibility has been obtained from demyelination models examining specific gangliosides, including GD1a (117–119).

#### 2.4.3 Downstream cellular injury and myelin repair

Immune activation and disrupted sphingolipid metabolism may jointly contribute to oligodendrocyte apoptosis, demyelination, and axonal injury. α2-3-GSLs may influence these processes by modulating inflammation, cell death, and the myelin microenvironment (**Figures 6A, B**).

At the level of cellular injury, the accumulation of ceramide and sphingosine may exert cytotoxic effects. Sphingosine can initiate oligodendrocyte apoptosis through the apoptosis antigen 1/Fas cell-surface death receptor (APO-1/Fas) pathway (108, 120). Complement activation and proinflammatory cytokine release may also increase cellular sensitivity to apoptotic signals and thereby aggravate myelin and axonal damage (121, 122). Thus, modulation of upstream inflammation and sphingolipid metabolism may help reduce oligodendrocyte death and myelin loss.

At the repair level, S1P–S1PR1/S1PR5 signaling may regulate oligodendrocyte progenitor-cell migration, differentiation, and maturation in a stage- and receptor subtype-dependent manner and may contribute to myelin maintenance and remyelination (117, 118). However, the available evidence derives mainly from *in vitro* studies, demyelination models, and studies of S1PR modulators; these effects therefore cannot yet be attributed directly to α2-3-GSLs.

Importantly, the roles of α2-3-GSLs in MS depend on cell type, disease stage, and the immune microenvironment. These lipids therefore cannot be classified simply as protective molecules, and their therapeutic value requires careful evaluation.

GM1 and GD1a contribute to myelin homeostasis and immune regulation but can also serve as target antigens in autoimmune attack (23, 107, 123). Clinically, anti-GM1, anti-GD1a, and related antibodies can be detected in the serum and cerebrospinal fluid of patients with MS, with reported positivity rates of approximately 20%–50%, depending on the assay and disease subtype. Some studies have also associated anti-GD1a antibodies with disease severity or progressive MS (123–125). In animal models, antibodies against GM1, GM3, GM4, and sulfatide have been detected in relapsing–remitting EAE (126). Regulation at the cellular level is more complex: GM1 and GD1a on activated T cells can participate in immunosuppression mediated by galectin-1 (Gal-1)/transient receptor potential cation channel subfamily C member 5 (TRPC5), but may also be recognized by autoantibodies (127, 128). Their net effects therefore depend on disease stage, cell type, and the local immune environment, and exogenous supplementation may have bidirectional consequences. Current functional studies are based largely on acute EAE and *in vitro* models, which do not fully recapitulate the long-term course of relapsing–remitting or progressive MS, and large longitudinal human studies are lacking (107, 129). Observed myelin protection or repair may reflect both direct prodifferentiation effects and secondary anti-inflammatory effects, whereas the proposed autophagy–lysosome mechanism is currently supported mainly by *in vitro* evidence (130). Moreover, prolonged high-dose supplementation may disrupt endogenous sphingolipid metabolism or elicit immune responses, and efficacy may diminish at later disease stages (108). The compositional control and long-term safety of mixed glycosphingolipid preparations require further evaluation (108, 129).

### 2.5 Guillain–barré syndrome: molecular mimicry, immune effector mechanisms, and neural injury

**Figure 7:**
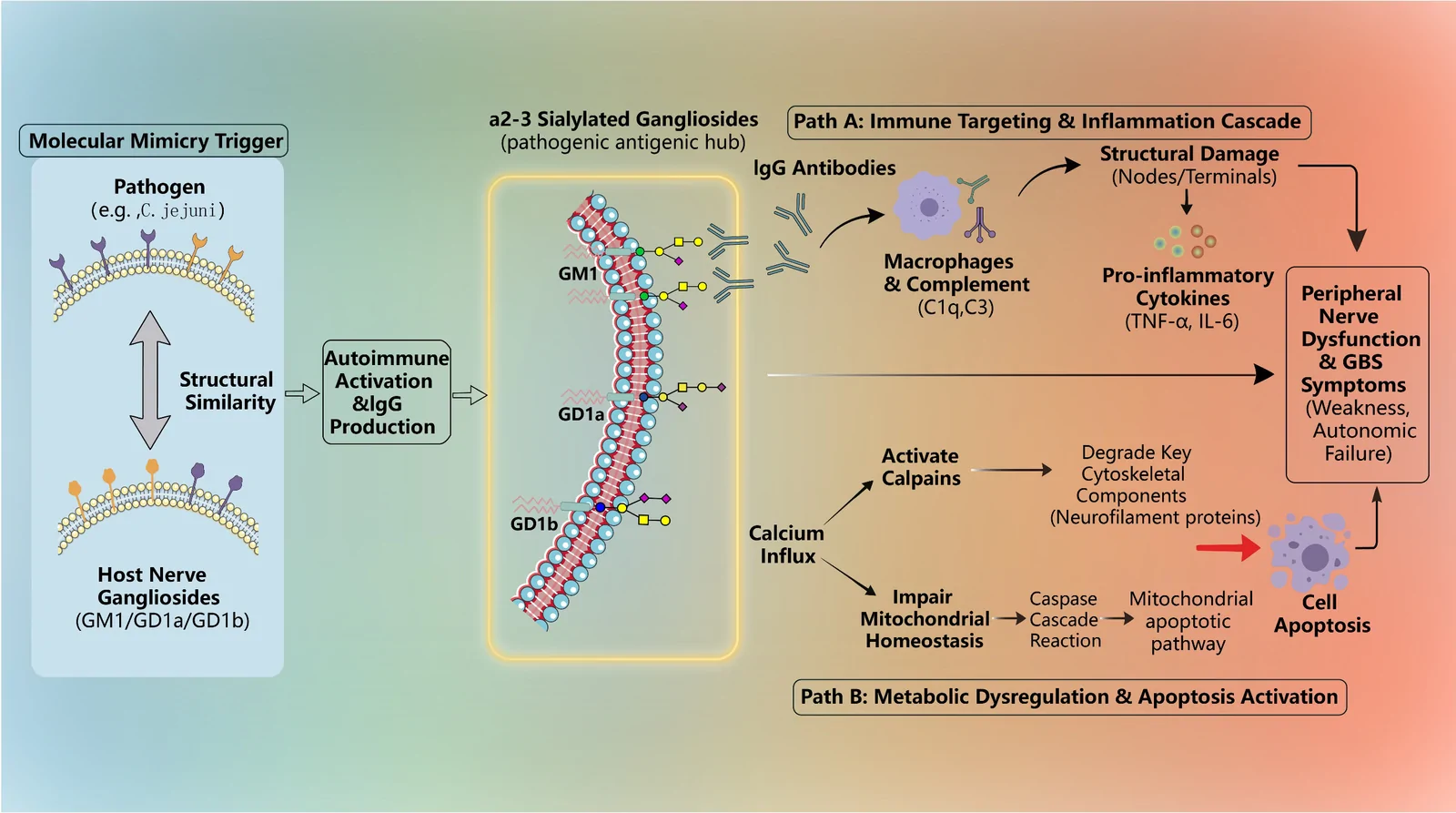
Evidence framework linking molecular mimicry, antiglycosphingolipid antibodies, complement, and axonal injury in selected GBS subtypes. Left, structural similarity between *C* jejuni LOS and host glycosphingolipids may elicit cross-reactive antibodies, although clinical onset is also influenced by host and immunoregulatory factors. **(A)** in a subset of antiglycosphingolipid antibody-positive axonal GBS cases and corresponding models, antibody binding can activate complement and promote neural injury. **(B)** model studies suggest that MAC-associated Ca^2+^ influx, calpain activation, and mitochondrial apoptotic signaling may contribute to axonal injury. This framework does not apply to all GBS subtypes and, in particular, cannot be directly generalized to AIDP.

GBS is an acute autoimmune disorder of the peripheral nervous system that typically manifests as rapidly progressive, symmetric limb weakness and autonomic dysfunction; severe cases may involve the respiratory muscles (131). Infection-associated molecular mimicry may elicit cross-reactive immune responses, after which complement activation, inflammatory-cell infiltration, and cellular injury may contribute to disease progression (30, 131–133). α2-3-GSLs such as GM1, GD1a, and GD1b are important membrane components of the peripheral nerve axolemma and nodes of Ranvier and may serve as target antigens for cross-reactive autoantibodies in GBS (132, 134). This section examines evidence at three levels: molecular mimicry, antibody-mediated effects, and downstream nerve injury. **Figure 7** primarily illustrates mechanisms relevant to antiglycosphingolipid antibody-associated axonal GBS and does not imply that all GBS subtypes follow the same pathway (133, 134).

#### 2.5.1 Molecular mimicry and initiation of the immune response

Campylobacter jejuni (C. jejuni) is a common antecedent pathogen in GBS. Its lipooligosaccharide (LOS) resembles α2-3-GSLs in peripheral nerve membranes and is considered an important basis for cross-reactive immune responses (**Figure 7**, left panel) (135, 136). GM1-like and GD1a-like LOS molecules may also form composite antigenic epitopes that mimic GM1b-associated glycolipid epitopes and induce anti-GM1b antibodies; antibody levels have been associated with disease severity in some patients with acute motor axonal GBS (137). N-glycolylneuraminic acid (Neu5Gc) in bovine-derived glycolipids is also immunogenic, although its actual contribution to GBS initiation remains unclear (28, 135). Animal studies further suggest that LOS dose may influence the immune outcome: low doses can induce TLR2/TLR4-independent immune tolerance, whereas higher doses may be more likely to elicit pathological immune responses (138).

The structural similarity between C. jejuni LOS and host α2-3-GSLs is supported by biochemical evidence, but molecular mimicry alone does not explain the complete disease process. Approximately 1 in 1,000 individuals with C. jejuni infection subsequently develop GBS (139), indicating that host factors also influence disease onset. Variation in human leukocyte antigen class II (HLA-II), immunoglobulin G Fc γ receptors (FcγRs), and complement-regulatory genes may affect the strength of cross-reactive immune responses, but no common susceptibility allele applicable to all GBS subtypes has been identified (140). Endogenous anti-idiotypic antibodies may also neutralize some cross-reactive antiglycosphingolipid antibodies and thereby limit clinical disease; this mechanism may contribute to the therapeutic effects of intravenous immunoglobulin (136, 141).

The role of Neu5Gc-modified glycosphingolipids in human GBS remains unclear. Because cytidine monophosphate-N-acetylneuraminic acid hydroxylase (CMAH) is inactive in humans, Neu5Gc cannot be synthesized endogenously; however, dietary exposure can induce low-titer natural anti-Neu5Gc antibodies in some individuals (142, 143). Whether these antibodies enhance cross-reactive immune responses or facilitate the clearance of exogenous glycolipids has not been established because evidence from *in vivo* experiments and clinical cohorts is lacking.

#### 2.5.2 Antibody-mediated immune injury

Antibodies against α2-3-GSLs show specific patterns of association with clinical GBS subtypes. Acute motor axonal neuropathy (AMAN) is frequently associated with antibodies against GM1, GM1b, GD1a, and GalNAc-GD1a (144, 145). In relevant experimental models, these antibodies can bind to the axolemma at the nodes of Ranvier, activate complement, and promote axonal injury (146). By contrast, high-titer IgG anti-GM1 antibodies are less frequently detected in acute inflammatory demyelinating polyradiculoneuropathy (AIDP) (147). IgG anti-GD1b antibodies have also been reported in Miller Fisher syndrome (MFS), in which they may affect cranial nerve terminals through complement-dependent mechanisms (148–150). Some studies have detected antibodies against GM1-containing glycolipid complexes across several electrophysiological subtypes, although positivity rates and reactivity vary (144, 145). These antibodies may therefore contribute to inflammation and neural injury in a subset of patients but should not be considered a single trigger common to all GBS subtypes (**Figure 7A**).

Individual glycolipid antibodies do not correspond simply to individual clinical subtypes. Ganglioside complexes (GSCs) formed by two or more glycosphingolipids within the same membrane microdomain, such as GM1–GD1a or GD1a–GD1b, may expose novel conformational epitopes. Antibodies against these composite epitopes may contribute to differences in the anatomical distribution of nerve involvement and electrophysiological phenotypes (151, 152) and may explain why conventional single-glycolipid ELISA is negative in some patients with axonal GBS (152).

Evidence for glycolipid antibodies in AIDP is comparatively limited, with positivity rates for conventional single-glycolipid antibodies below 15% (153). This finding suggests that AIDP may involve glycolipid–myelin-protein composite antigens or T-cell-mediated immune responses; autoreactive T cells targeting the myelin proteins P0 and P2 have been identified in patients with AIDP (133). Thus, the antiglycosphingolipid antibody mechanisms implicated in axonal GBS cannot be directly extrapolated to all GBS subtypes.

#### 2.5.3 Complement, calcium overload, and axonal injury

In antibody-mediated models of axonal injury, antibodies such as anti-GM1 can activate complement and induce formation of the membrane attack complex (MAC) after binding to antigens on neural membranes (154). MAC-induced membrane damage can promote Ca^2+^ influx (154) and increase the intracellular calcium load (155, 156). Elevated Ca^2+^ can activate calpain (157–159), leading to degradation of axonal cytoskeletal proteins such as neurofilaments (154). Concurrently, disrupted calcium homeostasis may impair mitochondrial membrane potential and promote opening of the mitochondrial permeability transition pore (mPTP), followed by cytochrome c release and activation of caspase-9/3-associated apoptotic signaling (160, 161). Together, these processes may contribute to axonal structural damage and dysfunction (**Figure 7B**).

## 3 Interactions of α2-3-GSLs with inflammation, immune responses, and programmed cell death

### 3.1 Structural basis and signal transduction

**Figure 8:**
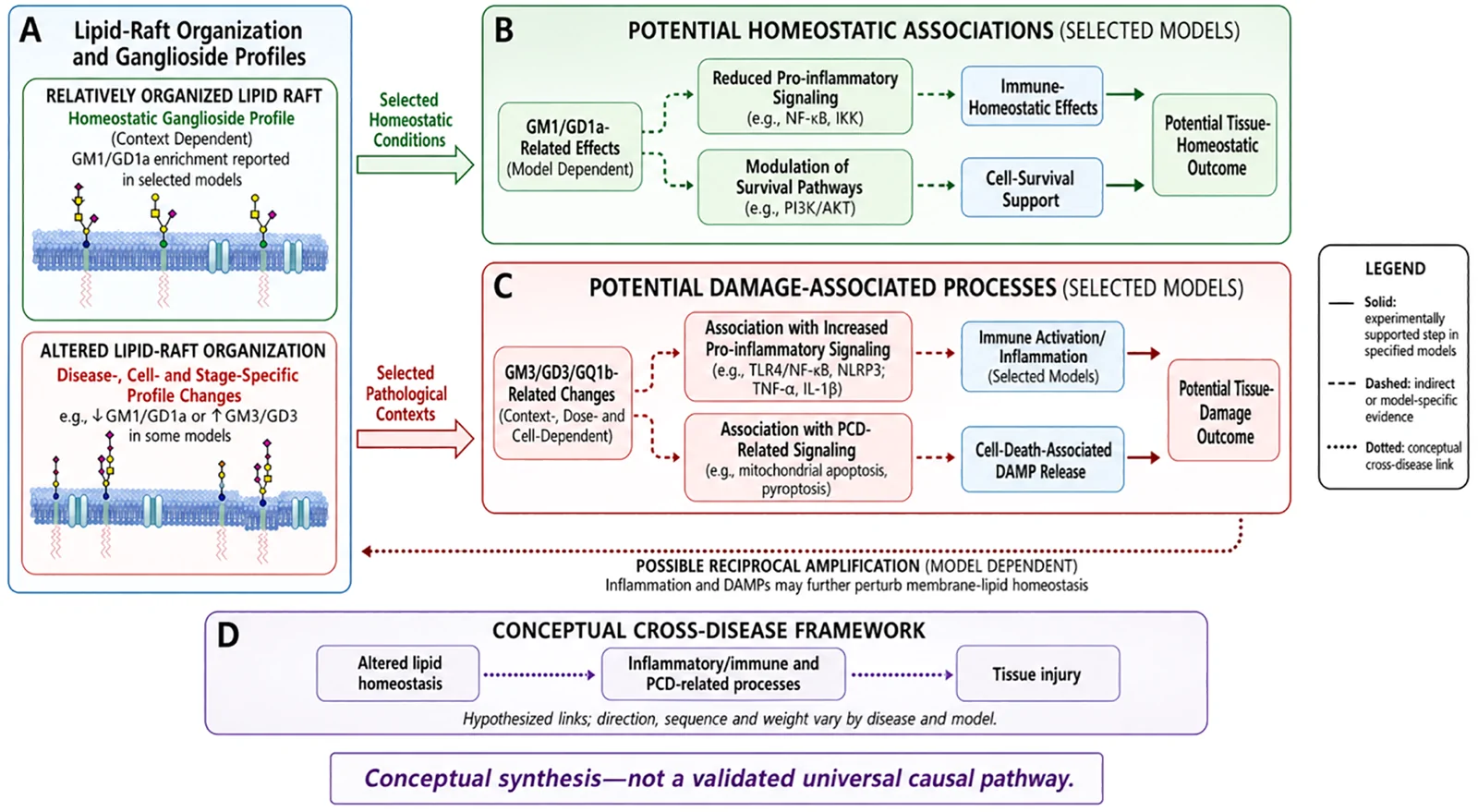
Conceptual integrative framework linking α2-3-GSLs, lipid rafts, inflammatory and immune responses, and programmed cell death. **(A)** Glycosphingolipids contribute to membrane-microdomain organization, and changes in their composition depend on disease, cell type, and disease stage. **(B)** In defined models, GM1/GD1a are associated with attenuated inflammation and modulation of survival-related signaling. **(C)** The effects of GM3, GD3, and GQ1b depend on concentration, cell type, and the pathological microenvironment; under certain conditions, they are associated with proinflammatory signaling, programmed cell death, and DAMP release. **(D)** A cross-disease conceptual framework synthesized from heterogeneous studies. Solid arrows indicate local steps supported experimentally in specific models, dashed arrows indicate indirect or model-specific associations, and dotted arrows indicate conceptual cross-disease links. The feedback relationships and integrated sequence shown do not represent a unified causal pathway validated across diseases.

Cell-membrane lipid rafts are dynamic nanoscale assemblies composed of glycosphingolipids, cholesterol, and functional proteins (162, 163). Exogenous stimuli or endogenous danger signals can promote the assembly of membrane receptors and neighboring molecules within lipid rafts, providing a structural basis for signal transduction (**Figure 8A**) (164, 165). The amphipathic structure of gangliosides contributes to lipid-raft organization and stability. Their hydrophobic ceramide tails interact with cholesterol and phospholipids and influence membrane thickness and fluidity (165), whereas α2-3-linked sialic acid residues in their hydrophilic glycans can form hydrogen bonds with other glycosphingolipids and the hydroxyl group of cholesterol and participate in cis or trans interactions (165). Gangliosides can also regulate the distribution of molecules such as epidermal growth factor receptor (EGFR), platelet-derived growth factor receptor (PDGFR), and tropomyosin receptor kinase A (TrkA) within membrane microdomains (166), thereby influencing downstream pathways, including mitogen-activated protein kinase (MAPK) and PI3K/AKT signaling (23, 84).

As an important ganglioside subclass, α2-3-GSLs share these structural features. Available studies suggest that changes in α2-3-GSL abundance in some diseases may accompany abnormal lipid-raft organization, but whether this represents a shared upstream event across diseases remains to be established. In specific models, gangliosides such as GM1 and GD3 can modulate the lipid-raft-dependent TrkA/Ras/extracellular signal-regulated kinase 1/2 (ERK1/2) pathway (164, 167). GM1, GQ1b, and other gangliosides may also affect the clustering of EGFR, PDGFR, T-cell receptors, and IgE receptors in raft domains and modulate downstream MAPK and PI3K/AKT signaling (23, 162). Differences in ganglioside glycan structure may further influence the selective binding and signal compartmentalization of molecules such as lymphocyte-specific protein tyrosine kinase (Lyn) and G proteins, thereby affecting pathway crosstalk (162, 168).

### 3.2 Context-dependent regulation of inflammation, immunity, and programmed cell death by α2-3-GSLs

#### 3.2.1 Context-dependent regulation of the inflammation–immunity axis

When the nervous system is exposed to exogenous or endogenous stress, different glycosphingolipid subtypes may exert opposing, context-dependent immunomodulatory effects through lipid-raft-dependent signal compartmentalization.

GM1/GD1a: In some models, these gangliosides predominantly exhibit anti-inflammatory effects (26). At the level of complement regulation, they can maintain the membrane localization and function of the complement-regulatory proteins CD55 and CD59, thereby limiting aberrant complement activation and the initial release of inflammatory mediators (28, 169, 170). At the signaling level, they can restrain excessive phosphorylation of IκB kinase (IKK) and p38 MAPK, reducing proinflammatory signal transduction (26, 171). These findings derive mainly from specific cellular and experimental systems, and their applicability across diseases requires further validation.

GM3/GQ1b/GD3: Their effects depend on concentration and the pathological context. At physiological concentrations, these glycosphingolipids contribute to membrane organization and cellular homeostasis. In some inflammatory models, higher levels are associated with enhanced innate immune-cell responses to danger signals, increased release of proinflammatory mediators, and infiltration of peripheral immune cells (26, 28, 172).

Under physiological conditions, different α2-3-GSL subtypes may collectively contribute to inflammatory homeostasis in the central and peripheral nervous systems (173). When the expression or function of this lipid family is altered—for example, when GM1/GD1a levels decline—their anti-inflammatory regulatory effects may be weakened, accompanied by activation of innate immune cells such as microglia, increased release of the proinflammatory cytokines IL-1β, IL-6, and TNF, and infiltration of peripheral immune cells into lesions (97).

The actions of α2-3-GSLs should not be reduced to a binary molecular switch. Their effects may vary continuously with concentration within membrane microdomains and may change direction under specific conditions, consistent with the dynamic phase-separation properties of lipid rafts (174).

At physiological concentrations, GM3 and GD3 contribute to normal neural-cell function. Physiological doses of GM3 can promote oligodendrocyte progenitor-cell maturation and myelin development in relevant models (175), whereas higher doses may inhibit cell survival (176). GD3 localized to MAMs has been linked to endoplasmic-reticulum–mitochondrial calcium transfer, autophagy, and energy-metabolism homeostasis (25, 177).

In some inflammatory models, increased GD3 and GM3 levels are associated with enhanced damage-related effects. GD3 can act on mitochondria and promote mPTP opening and cytochrome c (Cyt c) release in a concentration-dependent manner; this process can be inhibited by cyclosporin A (CsA) (178, 179). Experiments using purified mitochondria further support a direct effect of GD3 on membrane permeability (180). In addition, excessive accumulation of glycosphingolipids in lipid rafts may enhance TLR4 signaling and proinflammatory cytokine release in relevant models (181).

These concentration-dependent effects suggest that glycosphingolipid-targeted interventions should avoid indiscriminate inhibition or excessive supplementation and should instead aim to maintain lipid concentrations within the physiological range (179).

#### 3.2.2 Regulation of programmed cell death by inflammatory and immune responses

Under pathological conditions characterized by excessive adaptive immune activation or persistent sterile inflammation, α2-3-GSLs may regulate programmed cell death in a manner dependent on the specific lipid, pathway, and subcellular localization. In current models, GM1 is more commonly associated with antiapoptotic effects, whereas GD3 exhibits proapoptotic effects in some contexts.

GM1-associated antiapoptotic mechanisms: **Figure 8B** summarizes candidate protective mechanisms associated with GM1 in specific models. Available evidence suggests that GM1 may attenuate target-cell death related to excessive inflammatory or immune activation through several pathways. First, GM1 can activate TrkA and the PI3K/AKT survival pathway, promote phosphorylation of Bcl-2-associated agonist of cell death (Bad), and inhibit caspase-9 and caspase-3 activation, thereby reducing apoptotic signaling (84). Second, GM1 can restrain excessive NF-κB and p38 MAPK activation and reduce proinflammatory cytokine release, indirectly lowering the risk of neuronal or oligodendrocyte apoptosis (26). GM1 can also regulate intracellular Na^+^ and Ca^2+^ homeostasis and attenuate excitatory amino acid toxicity-associated injury (171).

GD3-associated programmed cell-death mechanisms: Unlike GM1, GD3 can promote programmed cell death in some models (**Figure 8C**). After translocation to the mitochondrial membrane, GD3 can promote mPTP opening, loss of membrane potential, and cytochrome c release, thereby enhancing mitochondria-dependent apoptosis (84). GD3 can also localize to autophagosomal membranes and colocalize with LC3, PI3P, and LAMP1, contributing to autophagosome formation and cargo processing (84). In some tumor models, GD3 can further promote apoptosis of CD8^+^ effector T cells through the endothelial differentiation-related factor 1/RelA/ST8 α-N-acetyl-neuraminide α-2,8-sialyltransferase 1 (EDF1/RelA/ST8SIA1) signaling axis (172). This finding should not be directly extrapolated to neurological diseases.

The involvement of GD3 in both autophagy and apoptosis suggests that its net effect may depend on subcellular localization. Abnormal vesicular sorting under pathological conditions may shift its role from homeostatic maintenance toward damage-associated effects, but this interpretation requires disease-specific validation (56, 81, 182).

#### 3.2.3 Feedback regulation of inflammatory and immune responses by programmed cell death

Pathological feedback: Programmed cell-death processes, including apoptosis and pyroptosis, can be accompanied by the release of IL-1β, IL-18, and DAMPs and by activation of innate immune cells such as microglia (183). Stimuli such as lipopolysaccharide (LPS) and IL-1β can also first induce microglial activation and proinflammatory cytokine expression. When inflammation persists, death of neural cells or oligodendrocytes may increase; the resulting cellular debris and nucleic acids can act as DAMPs to further activate TLR4/NF-κB signaling and promote local inflammation and infiltration of adaptive immune cells (**Figure 8C**) (26, 91). These events suggest a potential reciprocal amplification between programmed cell death and inflammatory–immune responses.

GM1-associated feedback regulation: GM1 may attenuate this feedback through two mechanisms. First, it may modulate the mode of cell death, reducing inflammatory forms of death such as pyroptosis and the accompanying release of DAMPs and proinflammatory mediators (183). Second, interactions between sialic acid residues in its glycan and inhibitory Siglec receptors on microglia may reduce the immune-cell response to cellular debris (26).

This regulation may depend on the mode of glycan–Siglec binding and the disease stage. Under physiological conditions, cis binding of microglial membrane glycans to Siglec receptors helps maintain a resting state, whereas extracellular glycosphingolipids released by apoptotic cells can influence this pathway through trans binding. In chronic degenerative diseases, disruption of lipid-raft organization may weaken cis masking and increase microglial activation (74, 75), potentially contributing to the limited efficacy of GM1 supplementation alone at advanced disease stages.

### 3.3 Potential shared pathological nodes associated with α2-3-GSLs across diseases

#### 3.3.1 Integration of disease-specific evidence

As discussed in Section 3.2.3, DAMPs and ganglioside-containing membrane fragments released by dying cells may promote microglial activation and complement responses and may enhance infiltrating immune-cell responses in some models of PD, AD, and MS; these effects may be further enhanced in the presence of antiglycosphingolipid autoantibodies (23, 144, 164, 169). Related studies also suggest that antibodies against glycosphingolipid complexes such as GM1–GD1a can produce stronger complement activation and tissue-damaging effects (144). Conversely, by maintaining lipid-raft, myelin, and axolemmal organization, GM1 and GD1a may reduce the release of cellular debris (23, 84) and dampen immune-cell responses to damage signals (23, 28, 171, 183); their deficiency is associated with amplified inflammation (171, 184). Disease-specific evidence is presented in Sections 2.1–2.5. Because these findings derive from different diseases and experimental systems, they should be regarded as supportive evidence for potential shared nodes rather than as a unified causal pathway (**Figure 8C**).

#### 3.3.2 Potential shared downstream pathological nodes and their boundaries of applicability

Although the initiating factors, affected cell types, anatomical targets, and immune mechanisms differ substantially among diseases, current studies suggest that, in selected diseases and experimental models, altered levels or distribution of α2-3-GSLs may be associated with downstream events such as disrupted membrane-microdomain organization, DAMP release, innate immune activation, and dysregulated programmed cell death (23, 84).

However, cross-disease causal studies using parallel designs are currently lacking; available studies have therefore not established that these events form a unified positive-feedback loop with the same direction and relative contribution in PD, AD, HD, MS, and GBS. The proposed sequence—disrupted lipid homeostasis, dysregulation of inflammatory/immune/cell-death networks, tissue injury, and further disruption of lipid metabolism—should therefore be understood as a conceptual analytical tool that integrates available observations, rather than as a fully established pathway shared across diseases.

In neurodegenerative diseases such as PD, AD, and HD, protein aggregation, abnormal lipid homeostasis, dysregulated cell death, and microglial activation may be interrelated. DAMPs released by injured cells can activate innate immune responses through pattern-recognition receptors and promote the production of proinflammatory mediators (17, 26, 185). In some central nervous system disorders, these changes may also be accompanied by infiltration of peripheral immune cells into lesions (186). By contrast, MS and GBS more prominently involve mechanisms such as autoimmune recognition, anti-ganglioside antibodies, and complement activation; α2-3-GSLs may also serve as target antigens in autoimmune attack rather than acting solely as homeostatic protective factors (105, 126–128). Thus, the temporal sequence, relative contribution, and net effect of these events may differ among diseases.

In summary, the integrative model proposed here may help organize potentially reciprocal amplifying relationships across diseases and provide a testable analytical framework for future studies. However, its completeness, cross-disease generalizability, and the causal relationships among its components require further validation (**Figure 8D**). Accordingly, future interventions should select specific targets according to disease type, disease stage, and the local microenvironment; neither restoration of membrane homeostasis nor glycosphingolipid supplementation should be considered a universally applicable therapeutic strategy (29).

## 4 Summary and outlook

Available evidence links several α2-3-GSL subtypes to inflammatory, immune, and programmed cell-death processes. Among them, GM1 has comparatively strong support for roles in lipid-raft organization and selected signaling events, although this evidence remains confined to particular experimental systems. Substantial heterogeneity across subtypes, disease models, and cellular contexts currently precludes treating α2-3-GSLs as a universal cross-disease regulatory hub. Future work should therefore first define the evidentiary boundaries and disease-specific constraints of the integrative model, then resolve dynamic interactions among subtypes, and finally use this knowledge to guide disease-specific interventions and clinical translation.

### 4.1 Conceptual boundaries, evidence tiers, and contextual constraints of the integrative model

The cross-disease regulatory network proposed in this review (**Figure 8D**) is a systems-level synthesis of otherwise fragmented experimental observations. Describing α2-3-GSLs as shared regulators across neurological disorders does not imply that this lipid family follows a single molecular pathway in different pathological environments. Existing studies vary widely in animal and cellular models and in the depth of mechanistic validation; interpreting the framework as an established mechanism would therefore overstate the evidence. The boundaries of the model are defined below in terms of evidence strength and disease-specific applicability.

To avoid assigning equal weight to findings supported at different levels, we classified the proposed mechanisms into three tiers according to direct causal validation, reproducibility across models, and disease-specific evidence.

Relatively well-supported evidence that remains experimentally constrained: Multiple cellular and animal models support the involvement of GM1 in lipid-raft organization, receptor-signaling compartmentalization, and selected cell-survival pathways. For example, GM1 may influence α-Syn- or mHTT-related toxicity in PD and HD models (39–43, 47) and has been associated with regulation of pro-survival signaling pathways, including PI3K/AKT (91). In BV2 cells, rodent models, and primary human microglia, GM1 has also been shown to suppress LPS-induced NF-κB hyperactivation and the release of proinflammatory mediators (26, 51). Collectively, these data support context-specific roles for GM1 in membrane homeostasis and inflammatory signaling, but they do not establish a uniform direction of effect across diseases, cell types, or disease stages (169, 171).

Mechanisms requiring further validation: Direct evidence that GM1/GD1a-mediated raft remodeling prevents TLR4 translocation into lipid rafts is derived mainly from PC12 cells and primary epithelial cells (52). Whether the same mechanism operates in microglia or constitutes a direct pathogenic or protective pathway in PD or other diseases remains untested in disease-relevant models. Evidence that GD3 localizes to autophagosomes or MAMs and contributes to autophagy regulation and mPTP opening comes primarily from fibroblasts, tumor cells, and acute-injury systems; subtype-specific knockout/rescue studies and long-term *in vivo* experiments in neurodegenerative disease models are still lacking (25, 57, 58, 172, 178–180). Similarly, although *in vitro* and structural biology studies partly support the hypothesis that Neu3-mediated hydrolysis converts GD1a to GM1, thereby providing a compensatory metabolic reservoir, the spatiotemporal dynamics and independent function of this process have not been directly demonstrated in disease-relevant contexts such as AD (82, 83, 86–88).

Conceptually inferred integrative model: The proposed positive-feedback sequence—disruption of lipid homeostasis, dysregulation of inflammatory/immune and cell-death networks, tissue injury, and further disturbance of lipid metabolism—is a conceptual synthesis of findings from disease-specific studies (23, 29, 84). To date, no causal study with a parallel cross-disease design has demonstrated that this sequence forms a complete loop with the same order and relative contribution across diseases. Accordingly, the model should be used as an analytical framework for organizing existing observations and generating testable hypotheses, not as a fully validated unified biochemical pathway.

The applicability of this conceptual framework is further constrained by disease type, disease stage, and the local pathological microenvironment. In protein-aggregation-associated neurodegenerative disorders such as PD, AD, and HD, abnormal protein aggregation is a major pathological feature. Changes in α2-3-GSLs may involve abnormalities in total abundance, subtype composition, or distribution within membrane microdomains and may influence neuron–glia communication, proteostasis, and inflammatory responses. However, the direction of these changes is not fully consistent across diseases or disease stages (35, 62–66, 90, 91).

The pathological contexts of the autoimmune neurological disorders MS and GBS also differ. In MS, gangliosides may help maintain myelin and immune homeostasis but may also serve as targets of autoimmune attack. In GBS, pathogen-associated molecular mimicry, anti-ganglioside antibodies, and complement-mediated peripheral nerve injury are more prominent (107, 108, 123–129, 131–134). The net effects of α2-3-GSLs should therefore not be interpreted as a simple switch between protective and pathogenic functions; they must be evaluated according to the specific subtype, cell type, antibody status, and disease stage. Defining these contextual constraints is essential for weighing the potential benefits and immunological risks of glycosphingolipid supplementation or targeted intervention and for guiding disease-specific mechanistic studies and drug development (108, 129). **Table 1** summarizes the evidence level, applicable context, and principal limitations of the mechanisms discussed above.

**Table 1:** Summary of α2-3-sialylated glycosphingolipid (α2-3-GSL) subtypes, molecular targets, associated pathways, observed effects, and evidence tiers across neurodegenerative and neuroautoimmune disorders.

Disease/research context	α2-3-GSL subtype(s)	Molecular/cellular target(s)	Associated pathway(s)	Principal observed effect(s)	Evidence type and experimental system(s)	Evidence tier and key limitation(s)	Direct evidence	Supporting evidence
PD: α-Syn conformation and aggregation	GM1 / OligoGM1	α-Syn monomers and early aggregates	Direct binding → α-helical stabilization → inhibition of fibrillization	Inhibits or delays α-Syn aggregation and in vitro toxicity	Cell-free biophysical assays, recombinant proteins, and in vitro models	Tier 1—Relatively well-supported direct molecular evidence; does not establish causality across the full course of human PD	(39, 48)	(35, 38)
PD: GM1 deficiency and phenotype	GM1 / OligoGM1	Nigrostriatal system, α-Syn pathology, and motor phenotype	Membrane lipid/protein homeostasis and neuroprotection	GM1 deficiency is associated with α-Syn aggregation and parkinsonian phenotypes; supplementation improves phenotypes	GM1-deficient mice, human PD tissue associations, and early clinical studies	Tier 1—Supported across multiple models and by human observational data; no unified clinical causal chain has been established	(40, 41, 43)	(38, 42)
PD: Autophagy-dependent clearance	Exogenous GM1	α-Syn, ATG13–ULK1, and the LC3 autophagy axis	Autophagy initiation → lysosomal degradation	Enhances autophagic flux, promotes α-Syn clearance, and reduces neurotoxicity	MPP^+^ cell and MPTP mouse models, including pharmacological inhibition of autophagy	Tier 1—Model-specific causal evidence; not yet validated in humans	(47)	(33, 58)
PD: α-Syn clearance in a human microglial model	OligoGM1	Intracellular α-Syn PFF burden and microglial activation state	Candidate phagocytic/autophagy–lysosomal mechanisms and inflammatory modulation	Reduces intracellular α-Syn burden and attenuates selected inflammatory phenotypes	Single HMC3 human microglial cell-line model	Tier 2—Preliminary evidence from a human cell-line model; neither TREM2 nor autophagy has been shown to be a necessary mediator	(45)	(26, 31)
PD-associated inflammatory context	GM1 / GD1a	TLR4 translocation to lipid rafts, Akt/TAK1, and NADPH oxidase	Lipid-raft-dependent TLR4/NF-κB inflammatory signaling	Attenuates LPS-induced inflammatory and oxidative-stress responses	In vitro PC12, epithelial-cell, and MG6 microglial models	Tier 2—Direct cellular evidence from non-PD-specific systems; not validated in α-Syn-induced microglial signaling	(51, 52)	(26, 50)
PD: Neurogenesis in an A53T model	GD3 + GM1	α-Syn/TH expression and olfactory-bulb neural stem/progenitor cells	Adult neurogenesis and potential proteostasis regulation	Combined treatment was associated with reduced α-Syn expression and improved olfactory-bulb neurogenesis	Combined intranasal administration in A53T α-Syn-transgenic mice	Tier 2—Preliminary in vivo evidence; effects cannot be attributed to either subtype individually, and direct GD3–α-Syn binding has not been demonstrated	(34, 44)	(53–55)
PD mechanistic extrapolation: Bidirectional effects of GD3	GD3	PI3P/LC3/LAMP1/MAMs; CD95/mitochondrial fission	Autophagosome formation and maturation; mitochondrial permeability and apoptosis	May support autophagic-membrane remodeling; abnormal accumulation may promote mitochondrial injury and apoptosis	Fibroblasts, T cells, and non-PD general mechanistic models	Tier 2—Direct mechanistic evidence derives from non-PD systems; the chronic in vivo net effect in PD remains extrapolative	(25, 56, 57, 60)	(58, 59, 61)
AD: Overall depletion and local GAβ enrichment	GM1 / GD1a; local GM1	GM1–Aβ binding and high-density GM1 raft clusters	Aβ conformational conversion, nucleation, and fibrillization	Local GM1 may promote Aβ aggregation despite overall GM1/GD1a depletion in the AD brain	Molecular/membrane models, in vitro studies, and human postmortem tissue	Tier 1—Relatively well-supported molecular-interaction evidence; the disease-level causal link between these spatial scales has not been established	(63–65)	(62, 66)
AD: Aβ–complement–synaptic injury	GM1/GAβ as a candidate upstream context	Aggregated Aβ, C1q/C3, and microglia	Classical complement pathway and complement-dependent synaptic engulfment	Complement deposition, inflammation, and early synapse loss	Biochemical, cellular, and AD mouse models	Tier 2—The Aβ–complement and complement–synaptic-loss links are relatively well supported; the GAβ–complement connection remains indirect and stage-dependent	(67, 71, 72)	(64, 68, 70)
AD-related homeostasis: Ganglioside-series deficiency	Complex ganglioside series	Complement system, microglia, and the inhibitory Siglec axis	Ganglioside loss → complement activation/immune disinhibition	Spontaneous complement activation, microglial proliferation, and neurodegeneration	GM2/GD2 synthase and GD3 synthase double-knockout mice	Tier 2—Moderate in vivo evidence; neither AD-specific nor attributable to GM1/GD1a alone; the Siglec interpretation is a supportive extrapolation	(73)	(74)
AD: GM1–autophagy–inflammasome axis	GM1	AKT/mTOR, autophagy–lysosome, and NLRP3	Enhanced autophagic flux and inflammasome suppression	Attenuates inflammation and promotes degradation of inflammation-associated proteins under AβO stimulation	Preprint using BV2 cells and primary microglia; related nanomodulation models	Tier 2—Preliminary evidence reported in a preprint (77); not yet established as mature causal evidence	(77)	(62, 78, 81)
AD: GD1a metabolic-reservoir hypothesis	GD1a → GM1	Membrane-bound neuraminidase 3 (Neu3)	GD1a desialylation and conversion to GM1	May provide local metabolic compensation for GM1 and indirectly affect lipid-raft/Aβ homeostasis	Biochemical and cell-membrane models; no AD-specific in vivo studies	Tier 2—Evidence from related mechanisms or models; spatiotemporal dynamics and independent function during AD progression have not been directly demonstrated	(83)	(63, 82, 84, 88)
HD: mHTT toxicity and pro-survival signaling	GM1	mHTT, PI3K/AKT, and HTT Ser13/Ser16	GM1 supplementation → AKT activation → HTT phosphorylation/pro-survival signaling	Reduces stress-induced apoptosis and mHTT toxicity; ameliorates neurodegeneration and behavioral deficits in HD mice	Intervention studies in HD cells, patient-derived fibroblasts, and multiple HD mouse models	Tier 1—Strong disease-specific preclinical evidence; no causal therapeutic evidence in patients; constrained by delivery and disease stage	(91, 92)	(84, 95, 98)
HD: Microglial innate immune tolerance	GM1	Innate immune tolerance in HD microglia	Inflammatory restimulation and development of tolerance	Partially corrects impaired innate immune tolerance in HD microglia	In vitro experiments in HD-derived/model microglia	Tier 2—Disease-relevant in vitro mechanistic evidence; lacks coculture and in vivo HD validation and is insufficient to establish a complete AKT–NF-κB cascade	(97)	(92, 98)
HD mechanistic extrapolation: GD3 and autophagy	GD3	LC3, PI3P, LAMP1, and autophagic membranes	Autophagosome formation and maturation; the link to mHTT clearance is speculative	May support autophagic flux, but evidence does not show that GD3 promotes mHTT clearance in HD	Non-HD human fibroblasts and general mechanistic studies	Tier 2—Conceptual extrapolation; no direct GD3–mHTT interaction or HD-specific in vivo knockout/rescue evidence	(25)	(99, 100, 102, 104, 106)
MS/EAE: Immune cytokine modulation	Brain-derived mixture (including GM1 and GD1a)	Th1/Th2/Th3 cytokine profiles	Regulation of adaptive immune polarization	Alters cytokine profiles and is associated with reduced EAE severity	EAE models and immunological experiments	Tier 2—Moderate preclinical evidence; effects of the mixed preparation cannot be assigned to a single subtype or directly extrapolated to efficacy in human MS	(109, 112)	(108, 111)
MS: Dual roles in myelin homeostasis and as autoantigens	GM1, GD1a, and related subtypes	MAG-mediated axon–myelin interactions and anti-ganglioside antibodies	Myelin structural stability; autoantigen recognition and potential humoral immune injury	Supports axon–myelin stability under physiological conditions; related antibodies are detectable in MS and EAE	Genetic/structural studies, patient serum/CSF, and multiple EAE models	Tiers 1/2—Substantial structural and associative evidence; antibody detection does not establish pathogenic causality and varies by disease subtype and assay	(110, 124, 127)	(23, 108, 131)
MS/EAE: Galectin-1-mediated immunoregulation	GM1	Galectin-1, TRPC5, and effector T cells	Gal-1-mediated GM1 cross-linking → TRPC5 activation → T-cell suppression	Suppresses effector T-cell proliferation and suggests potential attenuation of EAE	T-cell mechanistic and EAE-related experiments	Tier 2—Relatively direct cellular mechanism; disease evidence is limited to EAE and does not establish causality in human MS	(128)	(129)
GBS: Molecular mimicry in susceptible axonal contexts	GM1, GD1a, GM1b, and others	C. jejuni LOS and host ganglioside-complex epitopes	Structural mimicry and cross-reactive anti-ganglioside antibodies	May initiate cross-reactive immunity but is insufficient by itself to cause GBS	LOS structural/serological studies, human macrophage exposure, and clinical associations	Tiers 1/2—Substantial structural and immunological support; low penetrance and host genetic/immune regulation limit causal extrapolation	(138, 139)	(30, 132, 133, 136, 137, 140–142)
Anti-ganglioside antibody-positive axonal GBS: Antibody–complement effects	GM1, GM1b, GD1a, GalNAc-GD1a, and complexes	Axolemma at the nodes of Ranvier, classical complement, MAC, and calpain	Antibody binding → complement/MAC → Ca²^+^ influx → axonal cytoskeletal injury	Nodal disruption, conduction failure, and axonal degeneration	Patient serology/electrophysiology, complement assays, and GBS mouse models	Tier 1—Strong but subtype-specific evidence; supports an axonal mechanism and should not be generalized to all GBS subtypes	(145, 152, 155, 160)	(132, 134, 146, 147, 154, 156, 157, 162)
GBS: Ganglioside-complex epitopes and applicability limits in AIDP	GM1–GD1a, GD1a–GD1b, and other complexes	Novel conformational epitopes formed by cis clustering in lipid rafts; AIDP may preferentially involve myelin-protein/T-cell targets	Antibody recognition of complex epitopes and complement activation; subtype-specific immune pathways	Explains some negative single-ganglioside assays and clinical/electrophysiological heterogeneity	Patient serology, ganglioside-complex arrays, and complement assays	Tier 2—Moderate clinical-association and in vitro functional evidence; the antibody-mediated axonal GBS model should not be generalized to AIDP	(145, 152, 153)	(134, 146, 148, 154)
Potentially shared downstream nodes: Neurodegenerative disorders	α2-3-GSL family	DAMPs/PRRs, microglia, inflammatory mediators, and programmed cell death	Potential crosstalk among protein aggregation, lipid abnormalities, innate immunity, and cell death	Provides a framework for comparing candidate downstream nodes shared by PD, AD, and HD	Conceptual cross-study synthesis of independent disease models and reviews	Tier 3—Conceptual cross-study integration; no parallel cross-disease causal validation	—	(17, 26, 84, 186)
Potentially shared downstream nodes: Autoimmune neurological disorders	GM1, GD1a, GD1b, and related subtypes	Autoantigen recognition, anti-ganglioside antibodies, complement, and immune cells	Context-dependent conversion of homeostatic membrane components into immune targets	Provides a framework for comparing immune injury to myelin and the axolemma in MS and GBS	Conceptual cross-study synthesis of disease-specific animal models and clinical observations	Tier 3—Conceptual cross-study integration; MS and GBS have distinct initiating mechanisms, precluding a unified causal loop	—	(108, 124, 127, 132–134)

Evidence tiers: Tier 1, relatively well-supported evidence that remains constrained by the experimental system; Tier 2, incomplete, model-specific, or extrapolated evidence requiring further validation; Tier 3, conceptually inferred cross-study integration.

‘Direct evidence’ refers to studies that directly tested the target or mechanism specified in the corresponding row; it does not establish causality across the full disease course or a complete cross-disease causal chain. ‘Supporting evidence’ provides context from related mechanisms, models, or reviews.

### 4.2 Moving beyond single-subtype studies to define system-level regulation within the ganglioside family

Most studies have examined individual gangliosides in isolation, leaving the dynamic balance between cooperative and antagonistic effects among family members poorly characterized. This limitation is a major source of mechanistic fragmentation in the field. Future studies could combine spatial lipidomics with *in situ* glycomics to construct spatiotemporal maps of α2-3-GSLs throughout disease progression and address three priorities: competition for lipid-raft occupancy and dynamic interconversion among functional subtypes; disease-stage-dependent changes in the enzymes responsible for glycosphingolipid synthesis and catabolism; and the integrated effects of altered subtype ratios on downstream inflammatory and cell-death pathways.

To address the limitations of dynamic *in vivo* tracking, metabolic labeling could be integrated with *in situ* fluorescence and mass-spectrometry imaging. Such approaches would enable real-time observation of intracellular glycosphingolipid trafficking and help resolve the temporal and causal relationships within pathological cascades that cannot be captured by static ex vivo experiments (187).

### 4.3 Identifying structure-function-matched targets across heterogeneous diseases

Disrupted α2-3-GSL metabolism has been reported in central and peripheral neurological disorders, including PD, MS, and GBS. Nevertheless, common and disease-specific patterns have not been systematically compared, and the context-dependent functions of this lipid family remain incompletely defined.

Future mechanistic studies should determine how glycosphingolipid-dependent remodeling of lipid rafts may coordinately limit excessive complement activation, maintain inhibitory-receptor signaling at the immune-cell surface, and tune immune-cell sensitivity to damage-associated molecules (76). A central unresolved issue is the functional heterogeneity of these lipids: the same glycosphingolipid class may influence several forms of regulated cell death, including pyroptosis, autophagy-related cell death, and ferroptosis, while its net effect may differ substantially across diseases (59). This context dependence complicates broad-spectrum targeting but also provides an opportunity to develop disease-specific interventions. Longitudinal lipidomic profiling in large clinical cohorts will be important for distinguishing broadly protective candidates from highly disease-specific targets and for integrating currently fragmented observations.

### 4.4 Overcoming delivery and immunogenicity barriers for the clinical translation of subcellularly targeted therapies

Current glycosphingolipid-based interventions are limited by inefficient central nervous system delivery, rapid peripheral clearance, and off-target effects. Translational research should prioritize subtype-selective small-molecule modulators and exosome-mimetic nanocarriers (188, 189). Blood-brain barrier penetration remains a major challenge for central nervous system therapies, and intact gangliosides administered peripherally may not reach therapeutic concentrations within lesions. Combining receptor-mediated endocytosis-targeting modifications, nose-to-brain delivery, and targeted nanocarriers may improve drug accumulation in brain tissue while reducing peripheral exposure.

Interventions based on the lipid-raft integrity–glycosphingolipid homeostasis–transmembrane signaling axis should move beyond indiscriminate supplementation with exogenous lipids and instead aim to restore physiological lipid balance within lesions. Two priorities warrant particular attention. First, chemical modification of glycan chains to remove the non-human sialic acid Neu5Gc may reduce the risk that exogenous glycosphingolipids induce autoantibodies and aggravate demyelination (190, 191). Second, subcellularly targeted delivery could promote selective incorporation of glycosphingolipids into lipid rafts or prevent aberrant GD3 trafficking to MAMs, thereby potentially limiting reciprocal amplification among inflammation, immune injury, and programmed cell death. These strategies may provide a rational basis for more precise interventions in neurodegenerative and autoimmune neurological diseases.

In summary, progress in this field may shift therapeutic strategies from blocking isolated pathways toward restoring membrane homeostasis at the systems level. Defining how α2-3-GSLs connect lipid-raft organization, immune signaling, and cell fate could identify both shared and disease-specific intervention points and provide a membrane-lipid-centered framework for interpreting complex pathological networks.
